# MechBBB: A Two-Stage Mechanism Informed Machine Learning Tool for Blood Brain Barrier Permeability Prediction

**DOI:** 10.3390/ph19101524

**Published:** 2026-09-25

**Authors:** Yu Shin, Sahith Mada, Sivanesan Dakshanamurthy

**Affiliations:** 1 Department of Biomedical Engineering, University of Michigan, Ann Arbor, MI 48109, USA; 2 College of Computer, Mathematical, and Natural Sciences, University of Maryland, College Park, MD 20742, USA; 3 Lombardi Comprehensive Cancer Center, Georgetown University Medical Center, Washington, DC 20007, USA; 4 Innscite AI LLC, Herndon, VA 20170, USA

**Keywords:** blood brain barrier (BBB), BBB permeability prediction, CNS drug discovery, machine learning, mechanism informed modeling, P-glycoprotein efflux, solute carrier transporters, PAMPA permeability

## Abstract

**Background/Objectives:** Blood–brain barrier (BBB) permeability prediction is important for central nervous system drug discovery. Many models rely mainly on chemical structure, omit transport information, and may report optimistic performance when related compounds occur across training and test sets without adequate leakage control. We developed MechBBB, a two-stage, transport-informed machine-learning framework that adds learned efflux, influx, and passive-permeability scores to conventional molecular features and evaluates their added value under explicit leakage control. **Methods:** In Stage 1, three LightGBM models were trained on separate efflux, influx, and passive-permeability datasets to generate transport-related scores. BBBP compounds were excluded from Stage 1 training by InChIKey matching. In Stage 2, a LightGBM classifier was trained on BBBP using a Murcko scaffold split with the three Stage 1 scores, ten physicochemical descriptors, and 2048-bit ECFP4 fingerprints. Performance was compared with a descriptor-plus-fingerprint baseline without transport scores. External evaluation used a strict B3DB set (*n* = 4080) with no InChIKey or nonempty scaffold overlap with BBBP and was performed without retraining, recalibration, or threshold adjustment. MechBBB was also compared with official SwissADME BOILED-Egg predictions on a chemically matched external subset. **Results:** On the BBBP scaffold test set, MechBBB achieved an AUROC of 0.932, an AUPRC of 0.983, an MCC of 0.737, and a balanced accuracy of 0.866. The AUROC improvement over the descriptor-plus-fingerprint baseline was small but statistically significant in the paired test (ΔAUROC = 0.0096; *p* = 0.0425). Scaffold-grouped cross-validation did not show a consistent ranking advantage from adding the Stage 1 scores. On strict B3DB, MechBBB achieved an AUROC of 0.894 and an AUPRC of 0.893, with no significant ranking advantage over the baseline. On the matched B3DB subset (*n* = 4043), MechBBB achieved higher accuracy (0.814 vs. 0.682), balanced accuracy (0.806 vs. 0.693), sensitivity (0.910 vs. 0.541), and MCC (0.631 vs. 0.401) than SwissADME BOILED-Egg, whereas BOILED-Egg had higher specificity (0.845 vs. 0.703). SHAP analysis identified TPSA, NumHDonors, and p_pampa among the leading contributors. **Conclusions:** MechBBB provides a leakage controlled and externally tested framework that combines competitive BBB permeability prediction with per-compound transport-related information. The Stage 1 scores produced a modest internal improvement but did not provide a consistent external ranking advantage, indicating that their main added value is transport-related context rather than a universal increase in predictive accuracy. MechBBB can be used to prioritize compounds for CNS drug discovery before experimental BBB testing and identify compounds that may warrant follow-up studies of passive permeability or active transport. The MechBBB web tool is freely available.

## 1. Introduction

The blood–brain barrier (BBB) is a major challenge in central nervous system (CNS) drug development [1,2]. The BBB limits the movement of many small molecules into the brain and reduces therapeutic options for neurological disorders [3]. It is formed by brain endothelial cells with tight junctions and high transporter expression [2]. These cells express ATP-binding efflux transporters such as P-glycoprotein (P-gp), breast cancer resistance protein (BCRP), and multidrug resistance-associated proteins (MRPs) [4,5]. BBB entry can occur through passive transcellular diffusion or carrier mediated uptake by solute carrier (SLC) transporters [6,7]. P-gp and related efflux transporters can oppose brain entry by returning compounds to the bloodstream [8,9]. BBB permeability therefore depends on the combined effects of passive permeability, active efflux, and carrier mediated influx [7]. P-gp in particular limits brain penetration of many drug molecules, while molecular size, polarity, hydrogen bonding, and lipophilicity also influence brain exposure [10,11,12].

Machine-learning approaches to BBB prediction include Bayesian models and gradient boosting, while graph neural networks and chemical language models provide additional molecular representations [13,14,15,16,17]. Structure-based approaches generally use molecular representations and physicochemical descriptors [14,15]. Prior work by Cornelissen et al. explicitly compared passive diffusion, influx, and efflux, providing a mechanistic foundation for transport-informed prediction [18]. Physicochemical properties favorable for passive diffusion do not guarantee brain exposure when active efflux limits penetration [8,11]. Evaluation strategy also affects reported performance. Random splitting can produce optimistic estimates when structurally related compounds occur in both training and test sets, whereas scaffold-based evaluation provides a stricter assessment of generalization to different chemical series [15,19]. BBB benchmark datasets combine measurements from different experimental sources and commonly represent permeability as a binary endpoint, even though brain penetration is continuous and condition dependent [13,20]. These factors make leakage control, external validation, and cautious interpretation of small model differences important when evaluating BBB prediction methods [21,22].

Here, we present MechBBB, a two-stage machine-learning framework that incorporates learned transport-related scores into BBB permeability prediction. In Stage 1, separate LightGBM models generate efflux, influx, and PAMPA scores, denoted p_efflux, p_influx, and p_pampa, from auxiliary transport datasets. These values are learned transport-related outputs rather than experimental transporter measurements or transporter specific mechanistic probabilities. In Stage 2, the three scores are combined with physicochemical descriptors and ECFP4 fingerprints, and their contribution is evaluated against fingerprint-only and descriptor-plus-fingerprint baselines. All BBBP compounds were excluded from Stage 1 training by InChIKey, while Stage 2 used Murcko scaffold-based evaluation. External evaluation was performed on strict B3DB after removing InChIKey and nonempty Murcko scaffold overlap with all BBBP partitions, without retraining, recalibration, or threshold adjustment. This design was used to determine whether the auxiliary transport scores provide additional predictive information under controlled internal and external evaluation while also providing per-compound transport-related outputs through the MechBBB web interface.

## 2. Results and Discussion

MechBBB uses a two-stage framework that combines transport-related modeling with BBB permeability classification. In Stage 1, three LightGBM models generate efflux, influx, and PAMPA scores from separate auxiliary datasets [23]. In Stage 2, these scores are combined with 10 physicochemical descriptors and a 2048-bit ECFP4 fingerprint to predict BBB permeability [24]. The workflow also includes BBBP scaffold-based evaluation, strict B3DB external validation, SHAP-based interpretation, and deployment through the MechBBB web interface [20,25,26]. This design was used to evaluate whether transport-related scores provide additional information beyond conventional molecular features while maintaining explicit leakage controls between the auxiliary datasets and BBBP (Figure 1).

The workflow separates auxiliary transport score generation from the final BBB classification task. BBBP compounds were excluded from Stage 1 training by InChIKey, while Stage 2 used a Murcko scaffold split to separate training, validation, and test compounds [25,30]. The strict B3DB set was further filtered to remove InChIKey and nonempty Murcko scaffold overlap with all BBBP partitions, leaving 4080 compounds for external evaluation. Model development, isotonic calibration, and threshold selection were completed using BBBP only, and the frozen models were applied to B3DB without retraining, recalibration, or threshold adjustment. The following sections first describe the dataset characteristics and chemical space, then evaluate Stage 1 and Stage 2 performance, external generalization, calibration, model interpretation, error patterns, and benchmarking against existing BBB prediction approaches.

### 2.1. Dataset Characteristics and Chemical Space

The primary training dataset was the MoleculeNet BBBP benchmark [19]. After chemical standardization, conflict resolution, and deduplication, the dataset contained 1950 unique compounds, including 1491 BBB+ and 459 BBB− compounds. BBBP was divided by Murcko scaffolds into training, validation, and test sets using a target ratio of 70:15:15 [25]. The training set contained 1365 compounds, the validation set contained 292 compounds, and the test set contained 293 compounds. Compounds sharing the same nonempty scaffold were assigned to the same partition, while acyclic compounds with empty scaffolds were handled using singleton identifiers. B3DB served as the external evaluation resource and contained 7804 compounds before strict overlap filtering [20]. After removal of compounds overlapping the full BBBP dataset by InChIKey or nonempty Murcko scaffold, 4080 compounds remained for external evaluation. The resulting dataset sizes and class distributions are summarized below (Table 1), with the complete curation inventory and preprocessing procedures provided separately (Supplementary Tables S1 and S2; Supplementary Codes S1–S6).

The BBB+ fraction was 0.743 in training, 0.815 in validation, and 0.816 in test, showing that BBB+ compounds were more common than BBB− compounds across all three BBBP partitions. This class imbalance means that accuracy alone can give an incomplete picture of model performance because a classifier that predicts every compound as BBB+ could achieve relatively high accuracy while failing to identify any BBB− compounds [31]. AUPRC was used as the primary metric for hyperparameter selection because it summarizes precision and recall for the positive class, although its interpretation depends on class prevalence [32,33]. AUROC was also reported to describe ranking performance across both classes [34]. MCC was used for threshold selection because it incorporates all four cells of the confusion matrix and is informative when class sizes differ [31]. Balanced accuracy, sensitivity, and specificity were reported to describe classification performance for both BBB classes at the selected operating point [35]. The lower BBB+ fraction of 0.532 in strict B3DB makes these complementary metrics particularly important when comparing internal and external results.

Molecular weight, MolLogP, and TPSA were compared between the BBBP training set and the strict B3DB external set to examine differences in their physicochemical distributions (Figure 2).

Molecular weight showed a single peaked distribution in both datasets, with most BBBP training compounds falling between approximately 250 and 350 Da. The strict B3DB set showed a broader range and contained more compounds above 400 Da. MolLogP distributions were broadly similar, with both datasets showing substantial density between approximately 1 and 3. TPSA was more broadly distributed in strict B3DB and showed a higher median, indicating that the retained external population contained more polar compounds on average. These comparisons provide context for the external evaluation by showing that the two datasets differ in molecular size and polarity, as well as scaffold composition. The distributions are descriptive and do not establish that the filtering procedure alone caused the observed differences or that any single property explains changes in predictive performance. The property comparison was performed using the frozen descriptor and external evaluation records (Supplementary Codes S7 and S22).

The strict B3DB set provided a stronger overlap control than the earlier filter based only on BBBP training scaffolds. The filtering procedure began with 7804 processed B3DB compounds. InChIKey overlap with the full BBBP dataset removed 1768 compounds, followed by removal of 1426 compounds sharing nonempty training scaffolds, 168 sharing validation scaffolds, and 362 sharing test scaffolds. These steps left 4080 external compounds with no InChIKey overlap and no nonempty Murcko scaffold overlap with the full BBBP dataset. The external set also contained a greater proportion of BBB compounds than the BBBP training set, which affects interpretation of precision, AUPRC, accuracy, and threshold-dependent metrics [32]. The physicochemical distributions add context to these differences but do not show that performance changes arise from scaffold novelty, class balance, or chemical properties alone. The contribution of the Stage 1 scores and the generalization of Model C were therefore evaluated through the controlled ablation, scaffold grouped cross validation, paired statistical comparisons, and strict external results presented in the following sections (Supplementary Tables S7 and S16; Supplementary Code S12).

### 2.2. Chemical Space Visualization Using UMAP and PCA

UMAP and principal component analysis (PCA) were used to visualize the chemical space of the BBBP dataset using the 10 physicochemical descriptors and three Stage 1 transport scores [36,37]. Both methods used the same 13 continuous inputs and excluded the 2048-bit ECFP4 fingerprint. These analyses were performed for visualization and were not used to reduce the inputs supplied to the Stage 2 classifiers. UMAP provides a nonlinear projection that emphasizes local neighborhood structure, while PCA provides a linear representation of variation in the continuous input space [36,37]. The two approaches were used to examine how experimental BBB labels were distributed among compounds and where the test set errors from the frozen Model C appeared in the projected space. UMAP was first applied to the full cleaned BBBP dataset, and the held-out test compounds were displayed using their classification outcomes at the fixed threshold of 0.51. The resulting projections were generated from the frozen continuous feature records (Figure 3; Supplementary Code S19).

BBB+ and BBB− compounds overlapped across much of the UMAP space (Figure 3A). BBB+ compounds formed the majority of the dataset and were distributed throughout the main body of the projection. BBB− compounds appeared in many of the same regions and also gathered along a lower right arm. Both classes therefore occupied shared projected neighborhoods rather than forming a clean two-dimensional separation. Most test compounds were classified correctly at the fixed threshold, with true negatives concentrated along the lower right arm, false negatives appearing near that region and in the middle of the projection, and false positives distributed more toward the left and center (Figure 3B). No single well-separated region contained all errors. These observations show where the frozen model’s errors appear in the selected continuous feature space, but they do not identify their causes. UMAP emphasizes local structure and does not preserve all distances or relationships from the original input space [36]. Consequently, overlap or separation in this projection cannot be interpreted as a direct measure of model discrimination, scaffold generalization, or the biological contribution of individual transport scores [36].

PCA was used as a complementary linear visualization of the same 13 continuous inputs (Figure 4) [37].

The first two principal components explained 62.9% of the total variance, with 41.4% captured by PC1 and 21.5% by PC2. BBB+ and BBB− compounds overlapped across a broad region of the projection, while BBB− compounds extended further toward higher PC1 values. A smaller number of compounds appeared at the extremes of PC1 or PC2, indicating that the continuous descriptors and transport scores contained variation not fully represented by the central cluster. Unlike UMAP, PCA identifies orthogonal directions that maximize variance in the input data, but those directions do not necessarily correspond to the features most important for BBB classification [37]. The projection was therefore used to describe the distribution of the input space rather than to establish a decision boundary or determine which descriptors were responsible for the model’s predictions.

The PCA projection showed substantial overlap between BBB+ and BBB− compounds, consistent with the UMAP results and the absence of a simple separation in the selected continuous features (Figure 3 and Figure 4). The broader extension of BBB− compounds toward higher PC1 values indicates variation in the input space, but the projection alone does not identify the descriptors responsible for that direction or establish a biological explanation for the observed distribution. Likewise, overlap between classes does not imply that an individual descriptor or transport score lacks predictive value when combined with the remaining inputs. PCA and UMAP provide complementary descriptive summaries, while the contribution of each input to the trained classifier is examined through SHAP analysis [26]. Quantitative evidence for the predictive value of the Stage 1 scores comes from the Model B versus Model C ablation, scaffold grouped cross validation, and strict B3DB evaluation rather than from the visualizations alone. These distinctions are important because the projections describe the distribution of the selected inputs but do not establish causal mechanisms or independently validate the model’s ability to generalize to new scaffolds.

### 2.3. Comparative Performance of Model Variants on BBBP Test Set

Three model variants were compared on the held out BBBP Murcko scaffold test set to measure the contribution of each input group to BBB permeability prediction. Model A used ECFP4 fingerprints only [24]. Model B combined 10 physicochemical descriptors with ECFP4 fingerprints. Model C used the same descriptors and fingerprints as Model B together with the three Stage 1 scores: p_efflux, p_influx, and p_pampa. For each model, a decision threshold was selected on the BBBP validation set by maximizing MCC using calibrated probabilities, and the selected threshold was then applied without modification to the test set [31]. Ranking metrics were calculated from raw ensemble mean probabilities, while threshold dependent metrics were calculated from calibrated probabilities. This design allowed the three feature groups to be compared under a common model configuration while preserving the distinction between discrimination and classification at a fixed operating point. The resulting test performance is summarized below (Table 2), with complete metrics, compound level predictions, and the locked evaluation procedure provided separately (Supplementary Tables S8 and S9; Supplementary Code S12).

On the BBBP test set, Model C achieved the highest AUROC and AUPRC, as well as the highest MCC and balanced accuracy at its validation selected threshold. Model C achieved an AUROC of 0.932 (95% CI, 0.895–0.963) and an AUPRC of 0.983 (0.974–0.991), compared with 0.922 and 0.980 for Model B and 0.916 and 0.974 for Model A. The marginal confidence intervals overlapped, so they alone do not establish a consistent ranking between models [39]. A paired DeLong test using raw scores showed a small Model C versus Model B AUROC improvement of 0.0096 (95% CI, 0.0003–0.0189; *p* = 0.0425) [39]. The corrected paired AUPRC comparison also favored Model C, with ΔAUPRC = 0.0037 (95% CI, 0.0006–0.0079; *p* = 0.0122). These results support a modest improvement on the held-out BBBP test set, but they do not establish that Model C is uniformly superior across all datasets or evaluation protocols (Supplementary Table S10; Supplementary Codes S13 and S14).

Threshold-dependent performance also favored Model C in the revised evaluation. At the validation-selected MCC optimal thresholds, Model C achieved MCC of 0.737 (95% CI, 0.632–0.834) and balanced accuracy of 0.866 (0.805–0.921). Model B achieved MCC of 0.660 and balanced accuracy of 0.817, while Model A achieved MCC of 0.687 and balanced accuracy of 0.848. These metrics measure different aspects of performance because AUROC and AUPRC describe ranking across thresholds, whereas MCC and balanced accuracy depend on the selected decision threshold [31,33,34,35]. A model with better ranking does not necessarily achieve the highest MCC when each model is evaluated at its own validation selected threshold [34]. In the present test set, however, Model C achieved the highest values for both ranking and threshold dependent metrics. The paired Model C versus Model B differences in MCC and balanced accuracy were 0.0774 and 0.0484, respectively, with both comparisons yielding *p* = 0.0032. These results support the observed within test classification improvement while leaving the question of external generalization to the independent B3DB evaluation (Supplementary Table S10; Supplementary Code S13).

Confusion matrices were examined to show how the differences in threshold-dependent metrics arose from the four classification outcomes (Figure 5).

Each model was evaluated using its own threshold selected on the BBBP validation set, with Model A using 0.58, Model B using 0.42, and Model C using 0.51. The thresholds were not optimized using the test labels. This distinction is important because the selected threshold reflects the score distribution and validation performance of each model rather than a universal measure of model quality. The confusion matrices provide a more direct description of how false positives and false negatives contribute to MCC and balanced accuracy than the summary metrics alone [31,35]. They also allow the error tradeoffs between the fingerprint baseline, the physicochemical baseline, and the mechanism-augmented model to be compared on the same held-out compounds. The complete confusion matrix counts and threshold definitions are available in the frozen evaluation records (Supplementary Tables S6 and S9).

Model C produced 42 true negatives, 12 false positives, 11 false negatives, and 228 true positives at its primary threshold of 0.51 (Figure 5C). Compared with Model B, Model C reduced false positives from 17 to 12 and false negatives from 12 to 11, corresponding to six fewer total misclassifications. Model A produced 41 true negatives, 13 false positives, 15 false negatives, and 224 true positives, giving a different balance of errors at its own threshold. Model C therefore achieved the fewest false positives and false negatives among the three models on this particular test set, which is consistent with its higher MCC and balanced accuracy. The comparison is useful because the improvement was not limited to one class at the expense of the other under the selected operating points. However, these counts are specific to the held-out BBBP test set and the validation selected thresholds. They do not establish that the same reduction in both error types will occur on every external population, where class balance and score distributions may differ [40].

ROC and precision recall curves were examined to visualize discrimination across the full range of possible decision thresholds (Figure 6) [33,34].

Unlike the confusion matrices, these curves do not depend on a single operating point and therefore provide a complementary view of how the three models rank BBB+ and BBB− compounds. AUROC summarizes the relationship between true positive rate and false positive rate, while AUPRC summarizes precision and recall for the positive class [33,34]. The latter is particularly relevant to the imbalanced BBBP dataset, although its baseline and interpretation depend on the prevalence of BBB+ compounds [32]. All curves were calculated from the raw ensemble mean scores, with the five seed predictions averaged before metric calculation. This ensures that the plotted ranking results correspond to the AUROC and AUPRC values reported in Table 2 rather than to the isotonic calibrated scores used for classification. The curve generation and frozen metric records are provided separately (Supplementary Codes S12 and S23).

Model C reached the highest AUROC of 0.932 and AUPRC of 0.983, followed by Model B at 0.922 and 0.980 and Model A at 0.916 and 0.974, respectively (Figure 6A,B). The curves remained close across much of the operating range, consistent with the modest differences in the corresponding summary metrics. The improvement in ranking was therefore smaller than the improvement observed in MCC and balanced accuracy at the validation selected thresholds. This distinction is important because a small change in the ordering of compounds can affect classification outcomes when the decision threshold falls near a group of compounds with similar scores. The curves also show that no single operating point captures every possible screening goal [34]. A model may perform differently when the priority is to identify as many BBB+ compounds as possible or to reduce false positives among BBB− compounds. The reported AUROC and AUPRC values support a modest within test ranking advantage for Model C, while the confusion matrices and operating point analysis provide the corresponding classification context.

Three fixed operating points were evaluated for Model C to examine how sensitivity and specificity change under different screening goals (Table 3).

At the MCC optimal threshold of 0.51, Model C reached sensitivity of 0.954 and specificity of 0.778. This point balances the share of BBB+ compounds found against the share of false positives. At the high sensitivity threshold of 0.81, sensitivity was 0.900 and specificity was 0.815. This point fits a goal that holds high sensitivity while raising specificity above the primary threshold. At the high specificity threshold of 0.92, specificity rose to 0.926 and sensitivity fell to 0.703. This point fits a goal that keeps false positives low and accepts more missed BBB+ compounds. No single threshold fits every use case [34].

Model C was kept as the final MechBBB model. On the BBBP test set, Model C reached the highest AUROC, AUPRC, MCC, and balanced accuracy of the three variants. The AUROC gap over Model B was small and cleared the 0.05 mark in the paired DeLong test (Δ = 0.0096, *p* = 0.0425). The gap over Model B on the scaffold grouped cross-validation folds and on the strict B3DB external set was small and did not hold in one direction on every metric, so the ranking gain from the Stage 1 scores is treated as small rather than large. Model C also carries the three Stage 1 scores, which give a per-compound readout of predicted efflux, influx, and passive permeability behavior. These scores let users see the predicted transport behavior behind a call. The scores do not set the biological cause of BBB permeability for a single compound [41]. Model C was kept as the transport-informed model for prediction and for this per-compound readout. Model B maintains a strong physicochemical baseline, since the scaffold grouped cross-validation and the strict B3DB test did not show a steady discrimination gain for Model C over Model B.

### 2.4. Stage 1 Transport Model Performance and Score Behavior

The three Stage 1 transport models were evaluated using fair random and Murcko scaffold-based holdouts to examine how their performance changed when compounds with shared scaffolds were separated [25]. Both evaluations used the corrected leak safe datasets and matched training procedures, including the same feature representation, hyperparameters, class weighting, and early stopping protocol. The eligible datasets contained 2236 efflux compounds, 807 influx compounds, and 1442 PAMPA compounds after exclusion of all molecules overlapping BBBP by InChIKey. Each task was evaluated separately because the underlying labels represented different transport-related endpoints and had different class distributions. AUROC and AUPRC were calculated on the outer holdouts, and differences were defined as scaffold performance minus random performance. The random and scaffold holdouts contained different compounds, so their differences were treated as descriptive rather than paired statistical comparisons. Positive class prevalence was also considered when interpreting AUPRC because the baseline precision differs with the proportion of positive compounds [32]. The resulting performance values are summarized below (Table 4; Supplementary Table S13; Supplementary Code S8).

Scaffold-based testing lowered efflux AUROC from 0.8182 to 0.7639 and AUPRC from 0.8743 to 0.8284, representing the largest decrease among the three tasks. Influx AUROC declined slightly from 0.9463 to 0.9369, while PAMPA AUROC remained close to 0.900 under both splitting procedures. Influx AUPRC increased from 0.8539 to 0.9079, but the positive class prevalence also increased from approximately 0.130 in the random holdout to 0.278 in the scaffold holdout. This increase should therefore not be interpreted as a pure improvement in ranking performance [32]. The effect of scaffold splitting was task dependent, with efflux showing the greatest reduction in discrimination and PAMPA showing relatively stable performance. After evaluation, the Stage 1 models were refit on their complete eligible leak safe pools to generate the transport scores used in Stage 2. The corrected scores were then examined across the full cleaned BBBP dataset to assess their relationships with experimental BBB labels. These analyses describe learned transport-related signals rather than direct measurements of transporter activity or passive permeability (Figure 7; Supplementary Code S9).

The Stage 1 score distributions showed different degrees of separation between BBB+ and BBB− compounds. p_pampa showed the largest gap, with mean scores of 0.745 for BBB+ compounds and 0.342 for BBB− compounds, and corresponding medians of 0.922 and 0.174. This direction is consistent with the importance of passive membrane permeability in BBB penetration [7,42]. p_efflux showed the opposite pattern, with mean scores of 0.384 for BBB+ and 0.530 for BBB− compounds and medians of 0.347 and 0.566, respectively. p_influx showed a smaller difference and substantial overlap between classes, with means of 0.180 and 0.223 for BBB+ and BBB− compounds. Cohen’s d values were −0.652 for p_efflux, −0.193 for p_influx, and +1.228 for p_pampa, where positive values indicate higher mean scores in BBB+ compounds [43]. The largest marginal effect was therefore observed for p_pampa, followed by p_efflux, while p_influx showed a smaller effect. These distributions support an association between the auxiliary scores and BBB labels, but they do not establish the independent predictive contribution or biological mechanism of any individual score (Supplementary Table S14; Supplementary Code S20).

Pearson correlation analysis was used to examine how the Stage 1 scores related to the physicochemical descriptors already included in Stage 2 (Figure 8).

This analysis was essential because the auxiliary models were trained using the same ten descriptors and ECFP4 fingerprints that were also supplied directly to the final BBB classifier. A strong correlation between a transport score and a descriptor could indicate that the score captures some of the same chemical property trends, while a weak correlation would indicate only limited linear association with that particular descriptor [41]. Neither outcome alone establishes whether the score contributes new predictive information beyond the complete descriptor and fingerprint representation. The analysis therefore focused on describing the relationships between the three scores and the ten physicochemical properties rather than claiming statistical independence or mechanistic specificity. Pearson coefficients were calculated using the corrected Stage 1 refit scores paired with the cleaned BBBP descriptor table. The correlation matrix is shown in Figure 8, with the full numerical values provided (Supplementary Table S14).

p_pampa showed its strongest positive correlation with MolLogP (r = +0.687), consistent with the relationship between lipophilicity and passive membrane permeability [12,42]. It was also negatively correlated with TPSA (r = −0.653) and NumHDonors (r = −0.614), indicating that higher polarity and hydrogen bonding capacity were associated with lower learned PAMPA scores (Figure 8). p_efflux showed positive correlations with MolWt (r = +0.630), HeavyAtomCount (r = +0.629), and RingCount (r = +0.551). These associations indicate that the efflux score tracks several molecular size and structural properties but do not establish transporter-specific recognition mechanisms. p_influx showed weaker linear relationships with the descriptor set, with its largest absolute correlation observed for RingCount (r = −0.287), followed by MolLogP (r = −0.229). The corrected Stage 1 scores therefore showed class associated shifts of different magnitudes and partial linear overlap with the physicochemical descriptors. These descriptive results provide context for including the scores as additional Stage 2 inputs, but the Model B-versus-Model C ablation, scaffold-grouped cross-validation, and strict external evaluation provide the quantitative evidence for whether they improve BBB prediction.

### 2.5. Scaffold-Grouped Cross-Validation and Threshold Selection

Five-fold Murcko scaffold-grouped cross-validation was performed on the combined BBBP training and validation partitions (*n* = 1657) to examine whether the ranking performance observed in the held-out test set was stable across alternative scaffold groupings. Each fold was constructed so that compounds sharing the same scaffold group were not divided between the fitting and evaluation portions of that fold [19,25]. The held-out BBBP test set was excluded from this analysis. The cross-validation used the locked hyperparameters and was performed separately from the training-only Optuna search. It was not used to select new hyperparameters, calibrators, or decision thresholds. AUROC and AUPRC were calculated for each fold, and the mean and standard deviation across the five folds were reported. These standard deviations represent variation between scaffold folds rather than variation across ensemble seeds. The resulting performance values are summarized below (Table 5; Supplementary Table S15; Supplementary Code S11).

The five-fold scaffold-grouped cross-validation produced mean AUROCs of 0.874 ± 0.064, 0.892 ± 0.043, and 0.890 ± 0.043 for Models A, B, and C, respectively. Mean AUPRCs were 0.948 ± 0.027, 0.958 ± 0.013, and 0.956 ± 0.014. Models B and C therefore performed similarly, with Model B showing slightly higher mean AUROC and AUPRC across these folds. The differences were small relative to the observed fold variation, and the results did not demonstrate a consistent discrimination improvement from adding the Stage 1 scores. Model A showed the largest AUROC standard deviation, indicating greater variation in its ranking performance across the tested scaffold partitions. The mean AUROC of Model C was lower than its held-out BBBP test AUROC of 0.932, illustrating that performance can vary with the composition of the scaffold test partition. This comparison does not by itself establish that the held-out test result is an outlier, or that one model is uniformly preferable.

The mean and variability of the cross-validation results are visualized below (Figure 9).

The cross-validation results provide a complementary assessment of generalization beyond the single held-out BBBP test split. The close performance of Models B and C indicates that the three Stage 1 scores did not provide a steady ranking advantage across the tested scaffold groupings. This finding supports a cautious interpretation of the added feature block. The controlled ablation remains useful because Models B and C share the same underlying physicochemical descriptors, fingerprint representation, and locked training configuration, allowing the effect of the additional scores to be evaluated more directly than through comparisons with unrelated published models. However, neither the ablation nor cross validation can establish that the learned scores represent independent biological mechanisms [41]. Their predictive contribution must also be considered in the context of the strict B3DB external evaluation, where both chemical composition and class balance differ from BBBP. The combined evidence is therefore interpreted as a modest and evaluation dependent contribution rather than a universal gain in discrimination.

Isotonic regression was fitted separately for Models A, B, and C using only the BBBP validation set [29]. For each model, the five seed level probabilities were averaged first, and the isotonic mapping was then applied to the ensemble mean. Raw ensemble scores were retained for the primary AUROC and AUPRC analyses, while calibrated scores were used for threshold-dependent classification. The fitted calibrators were subsequently applied without modification to the held-out BBBP test set and strict B3DB external set. Primary decision thresholds were selected by maximizing MCC on the validation set, giving thresholds of 0.58 for Model A, 0.42 for Model B, and 0.51 for Model C. Two additional Model C operating points were selected at 0.81 and 0.92 to prioritize high sensitivity and high specificity, respectively. On the validation set, these secondary thresholds achieved sensitivity and specificity of 0.916 and 0.796, and 0.571 and 0.963. All thresholds were fixed before test evaluation, and no test or external labels were used to change them (Supplementary Table S6; Supplementary Code S11).

### 2.6. External Validation on Strict B3DB

Model performance was evaluated on B3DB as an external resource that was not used for Stage 2 model fitting, calibration, or threshold selection [20]. Before evaluation, B3DB compounds were filtered to remove molecular identity overlap with the union of all BBBP partitions and nonempty Murcko scaffold overlap with the BBBP training, validation, or test sets. The procedure reduced the processed B3DB dataset from 7804 compounds to 4080 strict external compounds, including 2170 BBB+ and 1910 BBB− compounds. The retained set therefore had a BBB+ prevalence of 53.2%, compared with 74.3% in the BBBP training set. All three models were applied without retraining, and the isotonic calibrators and thresholds selected using BBBP validation were carried over without modification. No B3DB labels were used to update the models or select new operating points. Raw ensemble mean scores were used for AUROC and AUPRC, while calibrated scores were used for threshold-dependent metrics. The resulting external performance is summarized below (Table 6; Supplementary Tables S7 and S16; Supplementary Code S12).

On strict B3DB, Model B achieved raw AUROC of 0.895 and AUPRC of 0.894, while Model C achieved AUROC of 0.894 and AUPRC of 0.893. Model A achieved AUROC of 0.883 and AUPRC of 0.887. At their fixed thresholds, Model C achieved the highest MCC of 0.634 and balanced accuracy of 0.808, compared with 0.630 and 0.803 for Model B. The ranking order therefore differed from the held-out BBBP test set, where Model C achieved the highest AUROC and AUPRC. The paired raw AUROC difference between Models C and B was −0.0008 (95% CI, −0.0026 to 0.0010; DeLong *p* = 0.374) [39]. The paired raw AUPRC difference was −0.0013 (95% CI, −0.0043 to 0.0017; *p* = 0.4058). Neither comparison established an external ranking advantage for Model C. The MCC difference was also small and not statistically significant, while the balanced accuracy difference was nominally positive at the 0.05 level. Taken together, the results support comparable external performance rather than uniform superiority of either model (Supplementary Table S10; Supplementary Codes S13 and S14).

The external results show that Model C retained strong discrimination under the strict overlap-controlled evaluation, despite differences in dataset source, scaffold composition, and class balance. However, the modest BBBP test improvement did not translate into a consistent ranking gain over Model B on B3DB. This distinction is important because the purpose of adding the Stage 1 scores was to provide transport-related information beyond the physicochemical and fingerprint baseline, not simply to increase the number of model inputs. The strict external evaluation indicates that the added scores can be incorporated without a substantial loss of external ranking performance, but it does not establish that they improve discrimination across every chemical population. The higher MCC and balanced accuracy of Model C at the fixed threshold should also be interpreted separately from its raw ranking metrics because the models use different validation selected thresholds.

To examine the sensitivity of classification performance to the operating point, the frozen Model C scores were evaluated across a range of thresholds without selecting a new external threshold (Figure 10).

The threshold robustness analysis showed that Model C classification performance varied across the evaluated threshold range. At the fixed primary threshold of 0.51, Model C achieved sensitivity of 0.909 and specificity of 0.708 on strict B3DB, corresponding to 1972 true positives, 1352 true negatives, 558 false positives, and 198 false negatives. The threshold was selected on BBBP validation, where the class balance and score distribution differed from those of B3DB. The external sweep therefore provides context for how a fixed operating point behaves after the transfer to a different evaluation population. The threshold producing the highest MCC on B3DB was not used to change the frozen classifier. The secondary thresholds of 0.81 and 0.92 illustrate alternative sensitivity and specificity tradeoffs, but their validation constraints are not guaranteed to hold on an external dataset [40]. These observations show that external classification performance depends on both model discrimination and the operating threshold carried over from BBBP. They do not establish that class balance alone caused the observed differences, and the raw AUROC and AUPRC results remain the primary measures of ranking performance.

A sensitivity analysis was performed to examine whether direct molecular overlap between strict B3DB and the Stage 1 training pools materially affected the external results. InChIKey matching identified 111 compounds in the strict external set that occurred in at least one Stage 1 training dataset. These compounds were removed, leaving 3969 compounds for the sensitivity analysis. The frozen Model C predictions, isotonic calibrator, and threshold were retained without retraining or adjustment. On the reduced set, calibrated AUROC was 0.897 and MCC was 0.650, compared with calibrated AUROC of 0.892 and MCC of 0.634 on the full strict B3DB set. Performance therefore remained similar after removal of the identified overlapping compounds, indicating that the observed external performance was not materially altered by this molecular overlap. The analysis does not establish complete assay independence or rule out other forms of dataset related bias, including shared source annotations or relationships not captured by InChIKey matching [21]. Full sensitivity results are provided separately (Supplementary Table S10; Supplementary Code S12).

### 2.7. Probability Calibration and Reliability Assessment

Isotonic regression was fitted on the BBBP validation set and applied without modification to the held-out BBBP test set and strict B3DB [29]. Calibration was assessed separately from discrimination because a model can rank compounds accurately while producing probabilities that do not match observed class frequencies [40]. Reliability diagrams were used to compare predicted probabilities with observed BBB+ fractions, while expected calibration error (ECE) summarized calibration discrepancy and the Brier score measured overall probabilistic prediction error [44,45]. Lower ECE indicates smaller estimated calibration discrepancy; a lower Brier score indicates better overall probabilistic accuracy, rather than calibration alone [40,44,45]. The validation reliability diagram was treated as a descriptive check because the calibrator was fitted on the same data, while the BBBP test set and strict B3DB provided independent evaluations of the frozen mapping. Raw ensemble mean scores were retained for the primary ranking analyses, and calibrated probabilities were used for classification at the fixed operating thresholds. The corrected reliability and score distribution results are shown below (Figure 11; Supplementary Tables S9 and S16; Supplementary Code S12).

On the held-out BBBP test set, isotonic calibration improved both ECE and Brier score (Figure 11A,B). ECE decreased from 0.0720 for the raw ensemble mean to 0.0482 after calibration, while Brier score decreased from 0.0822 to 0.0710. These results indicate that the validation-fitted mapping improved the agreement between predicted probabilities and observed BBB+ labels on the internal test population. The raw AUROC of Model C was 0.932 and the raw AUPRC was 0.983, while the corresponding calibrated values were 0.928 and 0.978. The modest reduction in ranking metrics is consistent with the fact that isotonic regression can introduce tied probability values and is not designed to optimize discrimination [29]. The calibrated score distribution nevertheless retained substantial separation between BBB+ and BBB− compounds, with an overlapping region in which classification depends on the selected threshold. The primary threshold of 0.51 was selected using validation MCC and was not adjusted to improve the test results. The internal calibration findings therefore support the use of the frozen mapping on BBBP test compounds while remaining distinct from evidence of calibration performance on an external population.

The same isotonic mapping transferred less well to strict B3DB (Figure 11C,D). ECE increased from 0.0488 to 0.1201, and Brier score increased from 0.1312 to 0.1495 after calibration. These results show that the validation-fitted adjustment worsened both calibration metrics on the external set despite the strong raw discrimination retained by Model C. The strict B3DB population differed from BBBP in class prevalence, scaffold composition, and physicochemical distributions, but the present analysis does not establish which of these factors caused the calibration shift. In particular, the observed deterioration should not be attributed solely to class balance or interpreted as proof that the isotonic method itself is unsuitable. No B3DB labels were used to refit the calibrator, preserving the independence of the external evaluation. The results therefore represent the performance of the frozen BBBP-derived probability mapping under external distribution change. They also indicate that calibrated probabilities should be interpreted cautiously when the model is applied to populations that differ substantially from the calibration data [40]. External recalibration could be examined in future work, but it was not performed in this study because it would alter the locked evaluation protocol (Supplementary Table S16).

### 2.8. Feature Importance and Interpretability

Mean absolute SHAP values ranked TPSA as the largest contributor at 0.735, followed by NumHDonors at 0.395 and p_pampa at 0.340 (Figure 12) [26,46].

MolWt ranked fourth at 0.174, followed by p_efflux at 0.168 and p_influx at 0.155. The prominence of TPSA and hydrogen bond donors is chemically consistent with the importance of polarity and hydrogen bonding in passive membrane permeability [11,47]. The high ranking of p_pampa also aligns with its substantial marginal separation between BBB+ and BBB− compounds. However, mean absolute SHAP values measure the magnitude of model contributions and do not by themselves establish whether a feature has a uniformly positive or negative effect on BBB prediction [26,46]. The individual contribution directions must be interpreted from the verified beeswarm distribution rather than inferred from feature importance alone. These results show that the model uses both conventional physicochemical information and learned transport related scores, while the controlled ablation remains necessary to assess whether the additional scores improve predictive performance beyond the baseline features (Supplementary Table S12).

The three Stage 1 scores contributed at different magnitudes within the fitted classifier. p_pampa was the highest ranked auxiliary score, followed by p_efflux and p_influx, indicating that the model assigned greater average attribution to the passive permeability score than to the other two transport related inputs. This finding differs from the earlier interpretation that p_influx was the dominant SHAP contributor, which is not supported by the corrected frozen analysis. The marginal class separation of p_influx was also smaller than that of p_pampa, with Cohen’s d of −0.193 rather than the obsolete near zero value. Marginal effect sizes and SHAP importance measure different quantities, so a smaller class difference does not necessarily imply that an input cannot contribute to a nonlinear classifier [26,43]. However, the present beeswarm analysis does not quantify explicit pairwise or higher order interactions, and it cannot establish that p_influx modifies predictions through a particular transporter mechanism. The results are therefore interpreted as evidence of learned model attribution rather than proof of interaction-driven biological effects. The predictive contribution of the auxiliary feature block was assessed through the controlled Model B versus Model C comparisons. Attribution and score behavior summaries are provided separately (Supplementary Tables S12 and S14).

#### 2.8.1. Fingerprint Input Analysis

Fingerprint inputs also contributed to Model C predictions, indicating that the classifier used structural information in addition to the continuous descriptors and Stage 1 scores. The ECFP4 representation consists of 2048 binary bits generated from circular atom environments using the Morgan algorithm at radius 2 [24]. Individual fingerprint bits can be ranked by their mean absolute SHAP values, but a bit index alone does not identify a unique chemical substructure [24,26]. In the corrected analysis, ECFP_bit_588 and ECFP_bit_967 were among the highest ranked fingerprint inputs, with mean absolute SHAP values of 0.148 and 0.147, followed by ECFP_bit_1928 at 0.118, ECFP_bit_80 at 0.114, and ECFP_bit_165 at 0.113. The full ranked feature list is provided separately (Supplementary Table S12). These values show that the fingerprint block contributes to the fitted model, but they do not establish that the identified bits represent specific functional groups or causal determinants of BBB permeability. A verified mapping from the frozen fingerprint bits to representative atom environments was not retained in the corrected interpretability outputs, so individual bit indices were not assigned chemical fragment structures in the revised analysis.

The absence of a verified bit mapping limits the chemical interpretation that can be drawn from the fingerprint importance results. Hashed fingerprints may map different atom environments to the same bit, and the same bit can be activated by different local structures across compounds [24]. A representative fragment therefore requires traceable information about the molecule, atom indices, radius, and fingerprint generation settings used to activate that bit [24,48]. Without these records, assigning a particular chemical structure to a ranked bit could give a misleading impression of specificity. The revised analysis consequently retains the numerical fingerprint importance results but does not use the earlier fragment panel as evidence of particular hydrophobic or polar substructures. The grouped SHAP summaries also indicate that the physicochemical, fingerprint, and Stage 1 blocks contribute to the model, although their summed importance values are affected by the different numbers of features in each block. These descriptive attributions do not establish that the fingerprint and auxiliary inputs provide statistically independent information. Their predictive contribution is assessed through the controlled model comparisons rather than through SHAP importance alone (Supplementary Table S12).

#### 2.8.2. Individual Compound Predictions

Global SHAP analysis describes the average contribution of each input across the BBBP test set, but the relative contribution of individual features can differ substantially between compounds. To illustrate this variation, three compounds were selected from the frozen BBBP test predictions using predefined criteria rather than selecting cases based on their SHAP patterns. The examples include a false positive with the highest calibrated probability among the false positive compounds, a false negative with the lowest calibrated probability among the false negative compounds, and a correctly classified BBB+ compound selected near the median calibrated probability of the true positive group. SHAP values were calculated from the five frozen Model C boosters and averaged across the ensemble using the same procedure as the global analysis [26]. The values are expressed on the raw LightGBM margin scale and therefore describe how each input shifts the model output before isotonic calibration. They should not be interpreted as direct contributions to the final calibrated probability or as evidence that an individual feature biologically caused BBB penetration (Figure 13) [41].

The three examples demonstrate that Model C does not base its classification on a single physicochemical descriptor, fingerprint bit, or Stage 1 score. In the false positive example, several inputs, including TPSA, ECFP bit 165, and NumHDonors, contributed positively to the raw margin, while MolLogP and several fingerprint bits contributed to the opposite direction. The combined positive contributions were sufficient for the compound to receive a high calibrated BBB+ probability despite its experimental BBB− label (Figure 13A). The false negative compound showed a markedly different pattern. TPSA, NumHDonors, ECFP bit 49, NumHAcceptors, MolWt, p_efflux, and p_pampa all contributed negatively to the raw margin, while only two of the displayed fingerprint inputs contributed positively, producing a strongly negative ensemble output and a calibrated probability of 0.000 (Figure 13B). In the correctly classified BBB+ example, positive contributions from TPSA, NumHDonors, p_pampa, p_influx, and several fingerprint inputs outweighed negative contributions from MolLogP, p_efflux, and other fingerprint features, resulting in a high BBB+ prediction (Figure 13C).

These case studies complement the global SHAP results by showing that the same input can contribute differently depending on the molecular context. For example, the contribution of a physicochemical property cannot be interpreted independently of the fingerprint and Stage 1 inputs because LightGBM learns nonlinear decision rules across the complete feature representation [23,49]. Similarly, the presence of p_efflux, p_influx, or p_pampa among the leading contributions for an individual compound does not establish that the corresponding transport process caused its experimental BBB behavior [41]. The false positive and false negative examples instead illustrate how combinations of structural, physicochemical, and learned transport-related inputs can produce incorrect classifications even when overall test performance is high. Experimental transporter measurements or matched compound series would be required to determine the biological explanation for an individual prediction.

### 2.9. Error Analysis and Failure Modes

Error analysis focused on the held out BBBP test set to examine the physicochemical regions associated with misclassification at the fixed Model C threshold of 0.51. Misclassifications consisted of false positives, defined as BBB− compounds predicted as BBB+, and false negatives, defined as BBB+ compounds predicted as BBB−. The frozen test predictions contained 12 false positives and 11 false negatives, together with 228 true positives and 42 true negatives among 293 compounds. Molecular weight and MolLogP were selected as reference axes because they represent molecular size and lipophilicity and are included among the physicochemical inputs used by the classifier. The distribution of correct and incorrect predictions was examined to determine whether errors were concentrated at extreme property values or occurred within regions also occupied by correctly classified compounds. This analysis was descriptive and was not used to select a new threshold, change model features, or modify any frozen predictions (Figure 14; Supplementary Tables S8 and S12).

Molecular weight and lipophilicity were used as reference descriptors because they influence passive membrane permeability and are related to several inputs used by the classifier [10,11]. Errors were not confined to the extremes of either axis, and many misclassified compounds appeared within the same broad property space as correctly classified compounds. This overlap indicates that molecular weight and MolLogP alone do not provide a simple boundary separating correct and incorrect predictions. Some errors appeared in intermediate property regions, where compounds with similar values on the two displayed axes could receive different classifications. However, the projection does not establish that these compounds are structurally close, that small fingerprint differences caused the errors, or that a particular transport or ionization process was responsible. BBB permeability reflects multiple physicochemical and biological factors, and the present model represents only a subset of those influences [2,7].

The error map therefore provides context for the limitations of the classifier without assigning causal explanations to individual compounds. The verified error counts are summarized below (Table 7).

The error groups showed different descriptive property profiles. False positives had median molecular weight and MolLogP values of 341.42 Da and 3.545, respectively, which were close to the corresponding true positive values of 335.01 Da and 3.513. Their median TPSA was higher than that of true positives, at 68.29 versus 47.06 Å^2^, while median p_pampa was lower, at 0.538 versus 0.944. False negatives showed higher median molecular weight and TPSA, at 427.46 Da and 112.74 Å^2^, together with lower median MolLogP of 1.847. Their median p_efflux was 0.746 compared with 0.384 among true positives, while median p_pampa was 0.202. These differences are consistent with the error groups occupying different regions of the selected feature space, but the distributions overlap and the numbers of false positives and false negatives are small. The results therefore describe associations within the test set and do not establish that any individual descriptor or Stage 1 score caused a misclassification.

The error analysis also illustrates why strong ranking performance does not eliminate errors at a fixed operating point. Model C achieved high AUROC and AUPRC on the BBBP test set, but 23 of the 293 compounds were misclassified at the validation-selected threshold of 0.51. These errors may reflect limitations in the molecular representation, incomplete transporter coverage, heterogeneous experimental labels, or chemical patterns that are not sufficiently represented in the training data [18,20,50]. The present analysis cannot distinguish between these possibilities without additional experimental evidence. In particular, the pooled influx score and P-gp-oriented efflux score do not identify the individual transporter responsible for the behavior of a compound. The error patterns shown should therefore be interpreted as descriptive evidence of model failure modes rather than mechanistic explanations. Additional transporter measurements, matched permeability assays, and validated uncertainty methods will be needed to determine why individual compounds are misclassified and to identify predictions that may be less reliable in future applications [51].

### 2.10. Benchmarking Against Published BBB Models

MechBBB performance was compared with published BBB classification results to provide context for its performance under scaffold-based evaluation. This comparison does not establish a definitive ranking because studies differ in dataset composition, preprocessing, label definitions, split construction, and model selection [22]. Random split results were not treated as directly comparable because structurally related compounds may appear in both training and test sets [15,19]. Published BBBP scaffold results are summarized alongside the present Model C results (Table 8).

Qin et al. reported BBBP scaffold AUROC values of 0.924 for MoleculeFormer, 0.916 for FP-GNN, and 0.886 for optimized Chemprop [52]. FP-GNN combines molecular fingerprints with graph-based representations [53], while Chemprop uses learned molecular representations for property prediction [15]. Model C achieved an AUROC of 0.932 on the held-out BBBP scaffold test set, together with MCC of 0.737 and balanced accuracy of 0.866, at its validation-selected threshold. These results provide context for MechBBB performance, but differences in preprocessing, test compounds, split construction, and model selection preclude a direct ranking [15,22]. The controlled comparison between Models B and C provides a more appropriate assessment of the effect of adding Stage 1 transport scores because both models were trained and evaluated under the same protocol.

On strict B3DB, Model C achieved raw AUROC of 0.894 and MCC of 0.634 after removing InChIKey and nonempty Murcko scaffold overlap against all BBBP partitions. This result provides a separate test of generalization beyond the dataset used for Stage 2 training, calibration, and threshold selection. It should not be directly compared with published BBBP scaffold values because strict B3DB differs in class balance, chemical composition, and experimental source. The external evaluation also showed that Model B and Model C had nearly identical raw ranking performance, with AUROC values of 0.895 and 0.894, respectively. The external results therefore do not establish a general discrimination advantage for Model C over the physicochemical baseline. Model C was retained as the final MechBBB model because it combines competitive predictive performance with explicit auxiliary transport-related outputs rather than because it uniformly outperforms all baseline or published methods (Supplementary Tables S9, S10, and S16).

#### Direct Comparison with SwissADME BOILED-Egg

A head-to-head comparison with the SwissADME BOILED-Egg method was performed to provide an additional reference benchmark under a shared external test population [54]. BOILED-Egg is a physicochemical method based on WLOGP and TPSA that provides a binary prediction of brain penetration [55]. Unlike MechBBB, it does not use structural fingerprints or the three Stage 1 transport scores [55]. Official SwissADME outputs were used rather than reconstructing the BOILED-Egg boundary locally [54]. The comparison was performed on the chemically verified matched subset of strict B3DB. Of the 4080 external compounds, 4043 had verified SwissADME outputs, including 2166 BBB+ and 1877 BBB− compounds. The remaining 37 compounds were excluded because of input length limitations, an unsupported lithium-containing structure, or unresolved identity mismatches. Frozen Model C predictions were evaluated using the fixed threshold of 0.51 without retraining, recalibration, or threshold adjustment, and both methods were compared against the same experimental labels (Table 9; Supplementary Table S11).

Model C achieved higher accuracy, balanced accuracy, sensitivity, and MCC than BOILED-Egg on the matched external set, while BOILED-Egg achieved higher specificity. Model C reached MCC of 0.631 compared with 0.401 for BOILED-Egg and sensitivity of 0.910 compared with 0.541. In contrast, BOILED-Egg achieved specificity of 0.845 compared with 0.703 for Model C, indicating that the two methods operate at different sensitivity and specificity tradeoffs. A paired McNemar analysis showed that 875 compounds were classified correctly by Model C and incorrectly by BOILED-Egg, whereas 344 compounds showed the opposite pattern, producing a statistically significant difference in paired classification performance [56]. However, this comparison evaluates the complete methods rather than isolating the contribution of the Stage 1 transport scores. MechBBB and BOILED-Egg differ in model architecture, feature representation, and decision rules, so the controlled Model B-versus-Model C comparison remains the more appropriate analysis for assessing the incremental contribution of the transport-related feature block (Supplementary Table S11).

A second comparison was performed on a predefined, class-stratified, structurally diverse panel selected independently of model predictions. Of the 100 selected compounds, 99 were successfully matched to verified SwissADME outputs, including 49 BBB+ and 50 BBB− compounds. On this panel, Model C achieved MCC of 0.365 compared with 0.196 for BOILED-Egg, but the paired difference was not statistically significant. The smaller panel was selected to emphasize structural diversity but contained substantially fewer compounds than the complete matched external set, limiting the precision of the comparison. The larger matched set therefore provides the stronger assessment of performance across the available strict B3DB population, while the diverse panel shows that a statistically significant advantage was not established across every sampled chemical subset. Together, these results indicate that MechBBB performed favorably relative to BOILED-Egg on the larger matched external dataset without supporting a claim of universal superiority (Supplementary Table S11).

### 2.11. MechBBB Web Interface

The MechBBB interface accepts SMILES strings or structure files and returns BBB permeability predictions using the full two-stage pipeline [57]. Single compound prediction and batch prediction are both supported. For single compound prediction, users enter a SMILES string or upload a structure file in SDF, MOL, PDB, PDBQT, or MOL2 format. For batch prediction, users upload a CSV file with a SMILES column and receive predictions for every row (Figure 15).

For each valid compound, MechBBB reports the raw ensemble mean, calibrated P(BBB+), binary classification, applied threshold, and three Stage 1 scores. The primary threshold is 0.51, selected by maximizing MCC on the BBBP validation set. Users may select the validation defined alternative thresholds of 0.81 and 0.92 or adjust the threshold for their screening objective. Threshold changes affect the binary classification without changing the predicted probabilities or Stage 1 scores. The Stage 1 scores are auxiliary transport-related outputs rather than measured transporter activities.

Prediction tables can be exported directly, including canonical SMILES, P(BBB+), binary BBB label, Stage 1 scores, and the applied threshold. A command line interface provides the same prediction functions for use in scripts and automated pipelines. The full project code is available at https://github.com/sivaGU/MechBBB (accessed on 22 September 2026). MechBBB is freely available as a web application at https://mechbbb.streamlit.app/ (accessed on 22 September 2026).

## 3. Materials and Methods

### 3.1. Dataset Collection and Quality Control

Five data resources supported model development and evaluation. The MoleculeNet BBBP dataset was used for Stage 2 BBB permeability classification, three transport datasets derived from the Cornelissen et al. compilation were used for Stage 1, and B3DB served as the external evaluation resource [18,19,20]. The auxiliary tasks represented efflux, influx, and passive membrane permeability. Dataset sizes and class distributions are summarized (Supplementary Table S1). The compound inventory and processing records are provided separately (Supplementary Table S2).

The Stage 1 datasets were obtained from the published Cornelissen supporting table rather than independently reconstructed from ChEMBL or Metrabase [18]. The source compilation incorporates transporter and permeability information from public resources, including ChEMBL and Metrabase, but the present study did not repeat the original assay extraction, text mining, or manual annotation procedures [18,58,59]. The ingestion pipeline used the source Status Efflux, Status Influx, and Status PAMPA fields as binary labels (Supplementary Code S2) [18]. Only records with usable molecular structures and endpoint assignments were retained after chemical standardization, conflict resolution, and deduplication. All BBBP compounds were subsequently excluded from the Stage 1 training pools by InChIKey matching against the union of the BBBP training, validation, and test sets [30]. The Figure 1 and Graphical abstract were created in Biorender.

#### 3.1.1. Efflux Dataset: P-gp

The efflux task used the binary Status Efflux annotations from the Cornelissen compilation [18]. The source publication describes P-gp-oriented efflux-positive compounds using an efflux ratio of at least 5 and nonsubstrates using a ratio of at most 1, with intermediate values excluded [18]. Metrabase-derived annotations for MDR1 and other ABC transporters were also incorporated into the source Status field [18,59]. The present pipeline consumed the curated binary status field and did not apply a new efflux ratio cutoff to raw assay measurements. The efflux annotations were used as a pooled P-gp-oriented transport endpoint. Because the ingested table did not retain complete assay level and transporter-specific provenance, the model was not treated as a gene-specific ABCB1 classifier. The resulting p_efflux score represents a learned efflux-related signal rather than a direct measurement of P-gp activity for an individual compound.

After standardization and deduplication, the processed efflux dataset contained 2457 compounds, including 1505 positive and 952 negative examples. InChIKey-based exclusion of 221 compounds overlapping BBBP left a leak safe pool of 2236 compounds, with 1390 positives and 846 negatives. The source label handling and exclusion counts are documented (Supplementary Table S2).

#### 3.1.2. Influx Dataset: SLC

The influx task used the curated Status Influx field from the Cornelissen compilation. The upstream annotations were assembled from transport data involving SLC22 and SLCO family members [18]. The present study did not independently extract transporter records from ChEMBL or Metrabase and did not impose a new uptake threshold of twofold above passive diffusion. The binary influx labels were pooled into one auxiliary classification task because the available data did not support reliable individual transporter models. As a result, p_influx represents a general uptake-related score and cannot identify the specific transporter responsible for a predicted signal. It should not be interpreted as a quantitative uptake rate or as evidence of activity at a particular SLC protein.

The processed influx dataset contained 886 compounds, including 157 positives and 729 negatives. Removal of 79 compounds overlapping BBBP left 807 leak safe compounds, with 134 positives and 673 negatives. The ingestion and exclusion records are provided (Supplementary Code S2; Supplementary Table S2).

#### 3.1.3. PAMPA Permeability Dataset

The passive permeability task used the Status PAMPA annotations from the Cornelissen compilation [18]. PAMPA measures transport across an artificial lipid membrane and provides a surrogate for passive membrane permeability without cellular transporters or active transport mechanisms [42,60]. It is not a direct experimental measurement of BBB penetration [42]. The source publication describes high permeability using Pe greater than 4 × 10^−6^ cm/s and low permeability using Pe less than 2 × 10^−6^ cm/s, with the intermediate range excluded [18]. The present pipeline used the curated binary status field rather than recalculating labels from raw Pe values.

After standardization and deduplication, the processed PAMPA dataset contained 1484 compounds, including 1235 high permeability and 249 low permeability examples. Exclusion of 42 BBBP overlapping compounds left 1442 leak safe compounds, with 1209 positives and 233 negatives. The source label provenance and processed dataset inventory are documented (Supplementary Table S2).

#### 3.1.4. B3DB External Evaluation Dataset

The Blood–Brain Barrier Database (B3DB) was used as an external source of experimental BBB permeability classifications [20]. The processed B3DB table contained 7804 compounds. It was not used for Stage 2 model fitting, hyperparameter optimization, calibration, or threshold selection (Supplementary Code S3). A strict external subset was constructed by removing any compound whose InChIKey occurred in any BBBP split and any remaining compound whose nonempty Murcko scaffold occurred in the BBBP training, validation, or test sets. The sequential filtering procedure reduced B3DB from 7804 to 6036 compounds after identity exclusion, to 4610 after training scaffold exclusion, to 4442 after validation scaffold exclusion, and finally to 4080 after test scaffold exclusion. The strict external set contained 2170 BBB+ and 1910 BBB− compounds, corresponding to a BBB+ fraction of 53.2%. The complete filtering record and final external set composition are provided (Supplementary Table S16).

The 4610-compound set was an intermediate filtering result and was not the primary external evaluation set for the corrected revision. The strict set had zero InChIKey overlap and zero nonempty Murcko scaffold overlap with the full BBBP dataset. Empty Murcko scaffolds were handled separately. The filtering procedure changes the class balance and chemical composition of B3DB, so the reported external performance applies to the retained strict subset rather than the entire database.

### 3.2. Curation, Preprocessing, Quality Control, and Labeling

SMILES strings were standardized using a common RDKit- based pipeline applied across BBBP, B3DB, and the Stage 1 transport datasets [27,48,57]. The pipeline parsed each SMILES string with RDKit, applied MolStandardize Cleanup, selected the fragment parent, and used Uncharger for charge normalization [48]. Canonical SMILES and InChIKeys were then generated from the standardized structures [30,57]. Molecules that could not be parsed or standardized were removed. Stereochemistry present in the input was preserved, and no additional stereoisomers were generated (Supplementary Code S4).

Duplicate and conflicting records were resolved at the InChIKey level [30,61]. When the same standardized molecular identity had conflicting binary labels within a dataset, all records associated with that conflicting identity were removed. Remaining duplicate records were reduced to one entry per InChIKey. This conservative procedure avoids assigning an arbitrary label to a compound with unresolved source disagreement [61]. The finalized BBBP dataset contained 1950 unique compounds from 2050 raw rows, including 1491 BBB+ and 459 BBB− compounds. The curation and compound inventory are summarized (Supplementary Table S2).

BBBP was divided into training, validation, and test sets using a Murcko scaffold-grouped greedy split with target proportions of 70%, 15%, and 15% and random seed 2026 [19,25]. Molecules sharing the same nonempty Murcko scaffold were assigned to the same partition. Acyclic compounds with empty Murcko scaffolds were assigned distinct singleton group identifiers based on their molecular identities, preventing all acyclic compounds from being treated as one artificial scaffold group. The split construction and scaffold utilities are provided (Supplementary Codes S5 and S6).

The resulting partitions contained 1365 training compounds, 292 validation compounds, and 293 test compounds. The corresponding BBB+ and BBB− counts were 1014/351, 238/54, and 239/54, respectively. The exact split membership was stored as InChIKey lists and reused throughout the corrected analysis. The training set was used for model fitting and hyperparameter optimization. The validation set was reserved for isotonic calibration and operating threshold selection, while the test set remained held out for final internal evaluation. The scaffold split does not enforce equal class prevalence, and the resulting class proportions were therefore reported alongside the performance metrics (Supplementary Table S1).

### 3.3. Feature Engineering

Molecular features were calculated from the standardized canonical SMILES using RDKit [48]. Two input blocks were generated for each molecule. The first contained 10 physicochemical descriptors, and the second contained a 2048-bit extended connectivity fingerprint (ECFP4). The descriptor block consisted of MolWt, TPSA, MolLogP, NumHDonors, NumHAcceptors, NumRotatableBonds, RingCount, HeavyAtomCount, FractionCSP3, and NumAromaticRings, in that fixed order. These descriptors summarize molecular size, polarity, hydrogen bonding, lipophilicity, and structural composition. TPSA uses fragment based polar surface contributions, MolLogP uses atom contributions, and FractionCSP3 summarizes carbon saturation [47,62,63]. The complete feature definitions and column order are provided (Supplementary Table S3).

ECFP4 fingerprints were generated using the RDKit Morgan fingerprint algorithm with radius 2, 2048 bits, and chirality disabled [24,48]. Each fingerprint was represented as a binary vector. The same feature generation functions and column order were used for Stage 1 and Stage 2 to prevent differences between training and inference (Supplementary Code S7). No descriptor scaling, zero variance filtering, or other feature selection procedure was applied to the classifier inputs. Molecules that failed feature generation were excluded during preprocessing. The resulting physicochemical and fingerprint blocks contained 2058 input dimensions. Stage 1 used these 2058 inputs, while Stage 2, Model C added three transport scores for a total of 2061 dimensions.

### 3.4. Stage 1 Transport Models

Three LightGBM binary classifiers were developed to generate the auxiliary efflux, influx, and PAMPA scores [23]. Each model used the 10 physicochemical descriptors and 2048-bit ECFP4 fingerprint. The three tasks were trained separately on their respective leak safe datasets. Their outputs, denoted p_efflux, p_influx, and p_pampa, were retained as continuous scores between 0 and 1 for subsequent use in Stage 2. The common fitting protocol and model parameters are provided (Supplementary Table S4).

All BBBP compounds were excluded from the Stage 1 training pools by InChIKey matching before model fitting [30]. The exclusion covered the union of the BBBP training, validation, and test partitions. This prevents direct molecular identity overlap between Stage 1 training data and the BBBP evaluation compounds. It does not establish complete assay independence or eliminate all possible relationships between the source datasets [21].

#### 3.4.1. Fair Random and Scaffold Based Evaluation

Stage 1 performance was evaluated using matched random and Murcko scaffold-based holdout protocols [15,25]. Each task was divided into an outer 80:20 training and holdout partition. For each protocol, a random 20% subset of the outer training partition was used only for early stopping. After the optimal boosting iteration was selected, the model was refit on the full outer training partition at that iteration and evaluated on the untouched outer holdout. The same feature representation, fixed hyperparameter block, class weighting rule, and evaluation procedure were used in the random and scaffold experiments (Supplementary Code S8).

Class imbalance was handled using scale_pos_weight = n_neg/n_pos, calculated from the corresponding training partition [23]. The fixed Stage 1 configuration included 48 leaves, maximum depth 5, a maximum of 5000 boosting rounds, and early stopping patience of 100. The remaining parameter values are recorded (Supplementary Table S4). Random seed 2026 was used for the evaluation. Empty Murcko scaffolds were assigned singleton identifiers so that acyclic compounds were not grouped together solely because their scaffold string was empty.

AUROC and AUPRC were calculated for the positive class on each outer holdout [33,34]. The random and scaffold holdouts contained different compounds, so their metric differences were treated as descriptive rather than paired statistical comparisons. Positive class prevalence was also considered when interpreting AUPRC [32]. The fair evaluation results and class distributions are reported (Supplementary Table S13).

#### 3.4.2. Final Stage 1 Refit and Score Generation

Each Stage 1 model was refit on its complete eligible leak safe pool to generate the transport scores used by Stage 2. The number of boosting rounds was fixed using the selected iterations from the scaffold-based early stopping runs. The final round counts were 520 for efflux, 538 for influx, and 637 for PAMPA. No held-out fraction was retained during these final refits (Supplementary Code S9).

The resulting models were applied to the complete, cleaned BBBP dataset to generate p_efflux, p_influx, and p_pampa for every Stage 2 compound. The scores were stored in the corrected final refit file, bbbp_mechanistic_probs_final_refit_v3.csv, and joined to the BBBP feature table by InChIKey. The Stage 2 evaluation lock records the use of this score file. The final refit models, rather than the models from the fair holdout experiments, were used for Stage 2 feature generation and deployment (Supplementary Code S10).

The Stage 1 outputs were not calibrated against their respective transport label prevalences. They are learned transport-related scores, not experimental measurements, quantitative transport rates, or calibrated probabilities of activity at a specific transporter [18,29]. A higher p_pampa indicates a stronger learned association with the high PAMPA class, while p_efflux and p_influx reflect their respective source label definitions. Their contribution to BBB classification was evaluated through the controlled Stage 2 ablation. Descriptive score distributions and correlations were generated separately (Supplementary Code S20; Supplementary Table S14).

### 3.5. Stage 2 BBB Classifier

The Stage 2 BBB classifier was trained only on the BBBP training partition (*n* = 1365). Three model variants were constructed to isolate the contribution of each feature block. Model A used the 2048-bit ECFP4 fingerprint alone. Model B combined ECFP4 with the 10 physicochemical descriptors, giving 2058 inputs. Model C, referred to as MechBBB, combined the Model B inputs with p_efflux, p_influx, and p_pampa, giving 2061 inputs. The feature matrices were assembled using the same fixed input schema (Supplementary Code S10; Supplementary Table S3).

The corrected Stage 1 scores were joined to BBBP by InChIKey before Stage 2 fitting. Median imputation for missing Stage 1 scores was implemented as a contingency, with each median calculated from the Stage 2 training partition only. In the corrected final refit score file, no Stage 1 values were missing, so no training, validation, or test compound required median imputation. No feature scaling or zero variance filtering was applied.

All three variants were trained as LightGBM binary classifiers using the same locked hyperparameter set [23]. Class imbalance was handled with scale_pos_weight = n_neg/n_pos, calculated separately for each fitting partition. Five model instances were trained using random seeds 0 through 4. For each instance, a nested random 20% subset of the training partition was used for early stopping. The selected boosting iteration was then used to refit the model on the full training partition. Neither the BBBP validation nor test labels were used for early stopping or tree parameter fitting (Supplementary Code S11).

The five seed-level predict_proba outputs were averaged to obtain one raw ensemble score per compound [64]. This raw score was subsequently transformed by the isotonic calibrator [29]. The final calibrated score, denoted P(BBB+), is the classification output used for threshold dependent decisions. The same training and ensemble procedure was applied to Models A, B, and C. Frozen model predictions and compound identities are provided (Supplementary Table S8).

### 3.6. Hyperparameter Optimization

Stage 2 hyperparameters were optimized using Optuna with the BBBP training partition only [28]. The search used the Model C feature block and five-fold Murcko scaffold-grouped cross-validation within the 1365 training compounds. GroupKFold was used so that no nonempty Murcko scaffold appeared in both the fitting and evaluation folds of the same split [65]. Acyclic compounds with empty scaffolds were handled as singleton groups. The optimization objective was the mean AUPRC for BBB+ across the five folds, calculated using average_precision_score [33,65]. A tree-structured Parzen estimator sampler was used with seed 2026 [28]. The optimization implementation and search protocol are provided (Supplementary Code S11; Supplementary Table S5).

The BBBP validation and test sets and B3DB were excluded from the hyperparameter search (Supplementary Table S5) [66]. A separate five-fold Murcko scaffold-grouped cross-validation was performed on the combined BBBP training and validation partitions (*n* = 1657) to assess ranking stability under alternative scaffold groupings. The held-out BBBP test set was excluded. This analysis used the locked hyperparameters and was not used for further tuning or threshold selection. Mean and standard deviation across the five folds were reported for AUROC and AUPRC. These standard deviations represent fold-to-fold variation, not variation across ensemble seeds. The cross-validation procedure and results are documented (Supplementary Table S15).

### 3.7. Calibration and Threshold Selection

The arithmetic mean of the five seed-level probabilities was used as the raw ensemble score for each Stage 2 model. Separate isotonic regression calibrators were fitted for Models A, B, and C using the BBBP validation partition (*n* = 292) [29]. The calibrators mapped raw ensemble scores to probabilities that better matched the observed BBB+ fractions in the validation data. Out-of-bounds clipping and output bounds of 0 to 1 were used. The fitted calibrators were applied without refitting to the BBBP test set and strict B3DB external set (Supplementary Code S11).

Primary AUROC and AUPRC values were calculated from the raw ensemble mean scores. Threshold-dependent metrics were calculated from calibrated probabilities. Calibration discrepancy was assessed using expected calibration error (ECE), while overall probabilistic prediction accuracy was assessed using the Brier score [44,45]. The validation reliability diagram was treated as a descriptive check because the calibrator was fitted on the same partition. Independent calibration assessment was performed on the held-out BBBP test set and strict B3DB. The calibration protocol and reported metrics are provided (Supplementary Table S6). Decision thresholds were selected using a fixed grid of 99 values from 0.01 to 0.99 in increments of 0.01. MCC, balanced accuracy, sensitivity, and specificity were calculated at each threshold [31,35]. The primary threshold for each model was the value maximizing validation MCC. Ties were resolved by higher sensitivity and then the lower threshold. The resulting thresholds were 0.58 for Model A, 0.42 for Model B, and 0.51 for Model C. The threshold selection rules and locked values are provided (Supplementary Table S6).

Two additional operating points were selected for Model C on the validation set. The high sensitivity threshold of 0.81 maximized specificity subject to sensitivity of at least 0.90. The high specificity threshold of 0.92 maximized sensitivity subject to specificity of at least 0.90. These thresholds represent alternative screening tradeoffs rather than confidence categories. The primary and secondary thresholds were fixed before test evaluation and were applied to B3DB without adjustment.

### 3.8. External Validation Protocol

Models A, B, and C were evaluated on the strict B3DB external set without retraining, recalibration, or threshold re-selection. The Stage 2 models, isotonic calibrators, and decision thresholds were fixed using BBBP only. No B3DB labels were used to update model parameters or select an operating point. The locked evaluation was implemented without modifying the frozen artifacts (Supplementary Code S12).

The strict external filter began with 7804 processed B3DB compounds. InChIKey overlap with the union of all BBBP splits was removed first, leaving 6036 compounds. Removal of 1426 additional nonempty training scaffold overlaps left 4610 compounds. Subsequent exclusion of 168 validation scaffold overlaps and 362 test scaffold overlaps produced the final strict set of 4080 compounds. The retained set contained 2170 BBB+ and 1910 BBB− compounds. Empty Murcko scaffolds were handled as singleton groups and were not treated as shared nonempty scaffolds. The final set had zero InChIKey and zero nonempty Murcko scaffold overlap with BBBP. The filtering protocol and external statistics are provided (Supplementary Table S7).

AUROC and AUPRC were calculated from raw ensemble mean probabilities [33,34]. Accuracy, balanced accuracy, sensitivity, specificity, precision, F1 score, MCC, and confusion matrix counts were calculated from calibrated probabilities at the fixed validation-selected thresholds [31,35]. Metrics were reported with class prevalence to support interpretation of performance across datasets with different class balances [32]. The complete BBBP and strict B3DB results are provided (Supplementary Tables S9 and S16).

#### 3.8.1. Statistical Analysis and Confidence Intervals

Marginal 95% confidence intervals for BBBP test and strict B3DB metrics were estimated using 10,000 class stratified bootstrap resamples with seed 2026 [38]. Interval endpoints were taken from the empirical 2.5th and 97.5th percentiles of the bootstrap distributions [38]. Class-stratified resampling preserved the positive and negative sample counts in each replicate (Supplementary Code S12).

Paired comparisons between Models B and C used predictions aligned by InChIKey on the same evaluation compounds. AUROC differences were assessed using a two-sided paired DeLong test for correlated ROC curves, with the difference defined as Model C minus Model B [39]. The analysis reported the paired difference, standard error, two-sided *p* value, and 95% confidence interval. Primary comparisons used raw ensemble mean scores (Supplementary Code S13).

Differences in AUPRC, MCC, and balanced accuracy were evaluated using paired class-stratified bootstrap resampling, with the same sampled compound indices applied to both models [38]. The corrected AUPRC analysis used average precision with tie-aware score handling and 10,000 bootstrap replicates [33,65]. Marginal confidence intervals and paired difference intervals were treated as separate quantities. The final corrected AUPRC confidence intervals and paired ΔAUPRC values are provided (Supplementary Code S14; Supplementary Table S10). The paired analyses were not used to modify the frozen models or select new thresholds.

#### 3.8.2. Stage 1 Overlap Sensitivity Analysis

An additional sensitivity analysis assessed whether direct overlap between strict B3DB compounds and the Stage 1 training pools influenced external performance. InChIKey matching identified 111 compounds in the strict external set that occurred in at least one Stage 1 training dataset. These compounds were removed, leaving 3969 compounds for the sensitivity analysis.

The frozen Model C predictions and validation-selected calibration and threshold were used without retraining or adjustment. Performance on this reduced set was compared descriptively with the full strict B3DB results. This analysis tested sensitivity to identified molecular overlap with the auxiliary datasets but did not establish complete assay independence or eliminate all possible source-related biases. The exclusion procedure and complete metrics are provided (Supplementary Table S16).

#### 3.8.3. Reference Benchmark Against SwissADME BOILED-Egg

A reference benchmark was performed using official SwissADME BOILED-Egg outputs [54]. BOILED-Egg is a physicochemical method based on WLOGP and TPSA that provides a binary prediction of brain penetration [55]. Its official exported classifications were used rather than reconstructing the model from a locally estimated boundary. Input preparation, batch generation, benchmark evaluation, and chemical identity reconciliation were performed using the released supplementary procedures (Supplementary Code S17).

The comparison was performed on a chemically verified matched subset of the strict B3DB external set. Input structures were prepared for SwissADME using the source chemical identities, and outputs were reconciled through submission identifiers and verified structures. Compounds with unresolved identity or classification mismatches were excluded rather than assigned substitute labels. The final matched set contained 4043 compounds, including 2166 BBB+ and 1877 BBB− compounds. Thirty-seven compounds were excluded, including 32 that exceeded the input length limit, one unsupported lithium containing structure, and four unresolved identity mismatches. The matching and exclusion records are provided (Supplementary Table S11).

The frozen Model C predictions were evaluated at the validation-selected threshold of 0.51. Accuracy, balanced accuracy, sensitivity, specificity, MCC, and paired McNemar tests were calculated on the shared matched compounds [56]. The paired test used the counts of compounds classified correctly by one method and incorrectly by the other. BOILED-Egg was evaluated as a binary classifier, and AUROC or AUPRC was not calculated from its binary labels (Supplementary Code S18).

A second comparison used a predefined, class-stratified, structurally diverse panel selected from strict B3DB using ECFP4-based MaxMin selection independently of model predictions [24,48]. Of the 100 selected compounds, 99 were successfully matched to verified SwissADME outputs. The same fixed model predictions and threshold were used. The panel analysis was reported separately because its chemical composition and sample size differed from the full matched external set. Neither benchmark was used to fit MechBBB or establish the independent contribution of the Stage 1 scores. The full panel results and compound identities are provided (Supplementary Table S11).

### 3.9. Feature Analysis and Error Analysis

SHAP analysis was used to examine the contributions of the physicochemical descriptors, fingerprint bits, and Stage 1 scores to Model C predictions on the held out BBBP test set [26,46]. The corrected analysis used TreeExplainer with tree path-dependent feature perturbation [26]. SHAP values were calculated for each of the five frozen LightGBM boosters and averaged across the ensemble. Values were reported on the raw model margin scale before probability transformation. They therefore describe additive contributions to the ensemble’s raw model output and are not exact additive decompositions of the isotonic calibrated probability (Supplementary Code S19).

Global feature importance was summarized using the mean absolute SHAP value across test compounds [26]. Beeswarm plots displayed both the magnitude and direction of individual feature contributions. The analysis was used to describe learned model behavior rather than establish biological causality. A positive or negative SHAP contribution indicates how an input shifts the model output relative to its reference expectation, not whether that input experimentally causes BBB penetration [41]. The global importance rankings are provided (Supplementary Table S12). No verified ECFP bit to fragment SMARTS mapping is claimed in the corrected release.

PCA and UMAP were used to visualize the continuous input space [36,37]. Both methods used the ten physicochemical descriptors and three Stage 1 scores for a total of 13 continuous inputs. The 2048-bit ECFP4 fingerprint was excluded from these visualizations. The continuous inputs were standardized using statistics fitted on the BBBP training partition, and the same transformation was applied to the validation and test compounds. PCA provided a linear projection, while UMAP was configured with 30 neighbors, minimum distance 0.3, and random seed 2026 [36,37]. Neither PCA nor UMAP was used for model fitting, feature selection, or calibration.

Stage 1 score distributions and correlations with physicochemical descriptors were analyzed on the cleaned BBBP dataset. These analyses were used to describe the marginal relationships between the auxiliary scores, molecular properties, and experimental BBB labels. They were not used to establish independent predictive contributions or biological causality. The descriptive analysis and associated summaries are provided (Supplementary Table S14).

Error analysis used the frozen Model C predictions on the BBBP test set at the primary threshold of 0.51. False positives were BBB− compounds predicted as BBB+, and false negatives were BBB+ compounds predicted as BBB−. Errors were examined in relation to molecular weight, MolLogP, TPSA, and Stage 1 scores, together with the PCA and UMAP projections. These analyses were descriptive and did not assign a causal explanation to individual errors. Case-level interpretations were restricted to compounds and SHAP values that could be reconciled with the frozen prediction and feature records (Supplementary Table S8).

### 3.10. Software and Reproducibility

Software environments, analysis scripts, and frozen model artifacts were documented to support inspection of the reported results and consistent prediction during deployment. The following subsections describe the research and deployment environments, preservation of evaluation artifacts, and checks performed to assess agreement between the deployed application and reference predictions.

#### 3.10.1. Python Environment and Software Dependencies

The corrected research pipeline was executed in Python 3.13.5 (Python Software Foundation, Wilmington, DE, USA; https://docs.python.org/3/ (accessed on 22 September 2026)) on Windows 11. The recorded numerical and data processing libraries were NumPy 2.3.1, pandas 2.3.1, and SciPy 1.16.2 (NumFOCUS, Austin, TX, USA) [67,68,69]. Model development used scikit-learn 1.7.2 (Inria, Palaiseau, France), LightGBM 4.5.0 (Microsoft Corporation, Redmond, WA, USA), and Optuna 3.6.1 (Preferred Networks, Inc., Tokyo, Japan) [23,28,65]. Chemical processing used RDKit 2025.03.3 (RDKit open-source cheminformatics project; Zenodo, Geneva, Switzerland), and interpretation used SHAP 0.48.0 (SHAP a common RDKit-based pipeline applied across BBBP) [26,48]. Plotting used matplotlib 3.10.6 (NumFOCUS, Austin, TX, USA) and seaborn 0.13.2 (seaborn open-source project), while dimensionality reduction used umap-learn 0.5.11 (umap-learn open-source project) [36,70,71]. The environment also included joblib 1.5.2 (joblib open-source project; Inria, Palaiseau, France; https://joblib.readthedocs.io/ (accessed on 22 September 2026)). These versions were taken from the corrected run environment snapshot rather than the older Python 3.9 requirements file. The recorded environment and dependency information are supplied with the Supplementary Code release.

The deployed Streamlit application used a separate Python 3.10 environment with RDKit 2025.03.3 and LightGBM 4.5.0 pinned for inference compatibility. The deployment dependency configuration was corrected after an earlier Cloud environment installed a different RDKit version and produced small raw score differences for two frozen reference compounds. No trained model or calibrator artifacts were changed during this repair.

#### 3.10.2. Rigor and Reproducibility

The corrected analysis is distributed as 27 numbered supplementary scripts. The package covers data ingestion, chemical standardization, scaffold splitting, feature generation, Stage 1 evaluation and refitting, Stage 2 optimization and training, calibration, locked evaluation, statistical analysis, SwissADME benchmarking, interpretability, and figure and report generation. The final code index records the purpose and provenance of each script and distinguishes the corrected release from the historical submitted scripts. The BBBP split membership, corrected Stage 1 score file, Stage 2 evaluation lock, model artifacts, calibration objects, and selected thresholds were retained as frozen outputs. In particular, the Stage 2 lock records consumption of the corrected final Stage 1 score file. Model evaluation was performed from these fixed artifacts rather than automatically retraining or recalibrating models when files were missing (Supplementary Code S12).

The final supplementary package also includes the corrected AUPRC bootstrap procedure (Supplementary Code S14). Source manifests and file hashes document the provenance of the packaged scripts. The packaging audit verified source integrity and syntax but did not establish a single end-to-end reproduction command that independently reruns every experiment. Reproduction instructions therefore distinguish between inspecting frozen outputs, running inference, and explicitly rerunning training or evaluation. The public GUI provides the frozen Model C inference pipeline. Its deployment was validated using an 18-compound reference fixture containing expected molecular identities, raw ensemble probabilities, calibrated probabilities, classifications, and available Stage 1 scores. After correction of the dependency environment, the public deployment matched all 18-reference raw and calibrated probabilities and classifications and all 24 available Stage 1 values within a tolerance of 10^−5^. The model artifacts and reference fixture were not modified during deployment validation.

### 3.11. Development of GUI for User BBB Prediction

A web-based graphical user interface named MechBBB was developed using Streamlit (version 1.38.0; Snowflake Inc., San Mateo, CA, USA; https://docs.streamlit.io/ (accessed on 22 September 2026)) to provide access to the frozen Model C classifier. The interface allows users to submit molecules without running the training pipeline or writing prediction code. The deployed application contains Home, Documentation, Demo Prediction Tool, and MechBBB ML Prediction pages. The demo page provides illustrative compounds, while the prediction page supports both single molecules and batch inference.

For single molecule prediction, users may enter a SMILES string or upload a supported structure file in SDF, MOL, PDB, PDBQT, or MOL2 format [57]. Structure files are parsed to obtain a molecular representation suitable for the same standardization and feature generation pipeline used during model development. Batch prediction accepts a CSV file containing a SMILES column and preserves the row order of the submitted file. Invalid or unparseable structures are reported with an error rather than assigned a valid BBB prediction. For valid molecules, the GUI calculates the ten physicochemical descriptors and -bit ECFP4 fingerprint, generates the three Stage 1 scores using the frozen auxiliary models, and combines these inputs into the 2061-dimensional Model C feature vector [24]. The five frozen Stage 2 boosters produce seed-level probabilities that are averaged before application of the fixed isotonic calibrator. The application reports the raw ensemble mean, calibrated P(BBB+), binary BBB classification, applied threshold, and Stage 1 scores. Canonical SMILES and InChIKey are included in the exported prediction records [30,57].

The primary decision threshold is 0.51. Users may select the validation defined high sensitivity and high specificity operating points of 0.81 and 0.92 or choose a user adjusted threshold. Threshold changes affect only the binary classification and do not alter the underlying raw or calibrated probabilities. The Stage 1 scores are displayed as auxiliary transport-related model outputs and are not represented as measured transporter activities or calibrated mechanistic probabilities. The fixed threshold definitions are provided (Supplementary Table S6). Molecular structures are displayed using RDKit-based two-dimensional rendering with a local SVG fallback when the standard drawing backend is unavailable [48]. The GUI does not require a molecular drawing service for the numerical prediction pipeline. The public application is available at https://mechbbb.streamlit.app/ (accessed on 22 September 2026), with source code and deployment materials at https://github.com/sivaGU/MechBBB (accessed on 22 September 2026).

## 4. Strengths

MechBBB incorporates transport-related information through Stage 1 scores that summarize efflux, influx, and passive membrane permeability patterns learned from separate auxiliary datasets. These scores are combined with structural fingerprints and physicochemical descriptors in a single BBB permeability classifier, allowing the contribution of the additional feature block to be evaluated against a common baseline. The controlled comparison showed that Model C achieved a modest improvement over Model B on the held-out BBBP scaffold test set, with raw AUROC increasing from 0.922 to 0.932 and MCC increasing from 0.660 to 0.737 at the respective validation-selected thresholds. Paired statistical analysis supported the small internal AUROC difference, while the strict external results did not establish a corresponding ranking advantage over Model B. This distinction is important because the value of the transport scores is not inferred solely from their biological interpretation or from overlapping separate model confidence intervals. The three auxiliary outputs also provide a transport-related readout alongside the final prediction, although they remain learned scores rather than direct measurements of transporter activity or explanations of the biological cause of BBB penetration.

A further strength is the use of explicit leakage controls and multiple evaluation settings. All BBBP compounds were excluded from Stage 1 training by InChIKey, and the auxiliary models were evaluated using matched random and Murcko scaffold-based holdouts before final refitting on their eligible transport pools. Stage 2 hyperparameter optimization was restricted to BBBP training data, while calibration and threshold selection used the validation partition. The held-out BBBP test set remained separate from these procedures. External validation was performed on 4080 B3DB compounds after removing InChIKey and nonempty Murcko scaffold overlap against the full BBBP dataset, including training, validation, and test partitions. No B3DB labels were used for retraining, recalibration, or threshold adjustment. The analysis also reports raw ranking metrics separately from calibrated classification metrics, includes paired uncertainty estimates, and examines the sensitivity of external performance to identified Stage 1 molecular overlap. These controls support a more transparent assessment of generalization than an internal random split alone, while the limitations of the auxiliary labels and external calibration are acknowledged rather than treated as resolved [21,22].

The study also provides a practical and reproducible implementation of the frozen prediction pipeline. The Supplementary Materials contain the dataset inventory, feature schema, compound level predictions, evaluation metrics, and supporting analysis procedures, allowing the reported results to be traced to their underlying records (Supplementary Tables S1–S16; Supplementary Codes S1–S27). The public GUI provides access to the trained Model C ensemble and reports both the final BBB prediction and the three auxiliary transport scores. A separate reference benchmark using official SwissADME BOILED-Egg outputs showed favorable performance for Model C on the chemically verified matched external set of 4043 compounds, although the predefined diverse panel did not show a statistically significant difference (Supplementary Table S11). This comparison provides context for the practical performance of the complete classifier but is not used as evidence that the Stage 1 scores independently improve prediction over Model B. Together, the controlled ablation, strict external evaluation, and accessible implementation support MechBBB as a screening tool with additional transport related outputs rather than as a universally superior BBB predictor.

## 5. Limitations

Several limitations should be considered when interpreting the results. Stage 1 training datasets may not cover the full range of structures in BBBP or B3DB, so Stage 1 scores may be less reliable for scaffolds that are sparse in the auxiliary training sets [18,50]. BBBP is class imbalanced, with BBB+ compounds making up approximately 74 to 82 percent of the scaffold partitions, and the modest test set size increases uncertainty in performance estimates. BBBP labels also reflect different assays and study conditions across literature sources, which introduces label noise that cannot be fully resolved during model training [13,20]. The binary labeling scheme simplifies a continuous and condition-dependent property, so compounds with different degrees of brain exposure may receive the same label [20,72]. Chirality was excluded from the ECFP4 fingerprint, which may limit prediction for compounds where stereochemistry influences BBB permeability or transporter recognition [73]. These factors should be considered when interpreting individual predictions and the generalization of the model.

A key limitation of the two-stage design is that the Stage 1 scores are learned outputs rather than experimental measurements of transport activity. The corrected Stage 1 models were evaluated using matched random and scaffold-based holdouts, and the efflux task showed the largest reduction in AUROC under scaffold evaluation. This indicates that the reliability of the auxiliary scores may vary across chemical scaffolds. The influx model also combines heterogeneous SLC uptake annotations into one label, so p_influx cannot identify the transporter responsible for a predicted signal. Similarly, the efflux model is P-gp oriented and does not include separate Stage 1 tasks for BCRP or MRPs, which also influence BBB entry [5]. Compounds whose permeability depends strongly on transporters not separately represented may therefore be insufficiently characterized. Although all BBBP compounds were excluded from Stage 1 training by InChIKey, complete assay independence and transporter-specific interpretation cannot be established from the available source records. The contribution of these scores should therefore be interpreted as a learned transport-related signal rather than a direct biological mechanism.

The incremental benefit of the Stage 1 feature block was modest and depended on the evaluation setting. Model C achieved a small, nominally significant AUROC improvement over Model B on the held out BBBP test set, but scaffold grouped cross validation showed similar performance, and strict B3DB did not establish an external ranking advantage. The strict external set removed InChIKey and nonempty Murcko scaffold overlap against all BBBP partitions, but this does not eliminate every possible source of dataset-related bias [21]. Isotonic calibration improved ECE and Brier score on the BBBP test set but worsened both metrics on strict B3DB, indicating that the validation-fitted probability mapping did not transfer equally well to the external population. Literature comparisons also depend on dataset composition, endpoint definitions, and split protocols, so reported values provide context rather than a direct ranking [22]. Finally, the current GUI does not provide a validated applicability domain assessment or per-compound uncertainty interval, and the model has not been prospectively evaluated on newly synthesized compounds. MechBBB should therefore be used as a screening tool rather than a substitute for experimental BBB permeability assessment.

## 6. Future Directions

Several directions could extend this work and address the limitations identified. Stage 1 coverage could be expanded by adding separate models for BCRP and MRPs to complement the current P-gp-oriented efflux signal [5,74]. Stage 1 influx could also be refined by modeling individual SLC transporters such as LAT1 and OATP1A2 rather than using a single pooled uptake label [6,75]. Larger and more varied transport training sets with clearer assay provenance could improve score reliability for scaffolds that are sparse in the current datasets [18,74]. Future Stage 1 models should also be evaluated under matched random and scaffold-based protocols to assess their generalization to new chemistry. Applicability domain methods could help identify compounds that fall outside the range of the auxiliary training data [50].

Uncertainty reporting could make screening decisions more informative when scores are near the operating threshold. Ensemble variation, conformal prediction, or other validated uncertainty methods could be examined to determine whether they identify unreliable predictions under external distribution shift [51,76]. External calibration could also be investigated using appropriately separated calibration data, since the current isotonic mapping did not transfer equally well to strict B3DB [40]. Regression on continuous endpoints such as logBB, Kp,brain, and Kp,uu,brain could preserve information lost in binary BBB labels and support medicinal chemistry by capturing gradations in brain exposure rather than a hard class boundary [11,72]. Such models would require consistent endpoint definitions and consideration of assay conditions, species, and total versus unbound concentrations [72].

External validation on newly synthesized compounds with matched assays would provide more direct evidence of performance in drug discovery [22,77]. Such studies could test whether the Stage 1 scores improve compound selection beyond physicochemical descriptors and fingerprints, particularly for compounds whose brain exposure is influenced by active transport. Literature comparisons could also be strengthened by evaluating baseline methods under the same preprocessing, scaffold splits, and overlap controls used here [15,22]. This would reduce differences caused by dataset composition and evaluation protocol and allow a more direct comparison of predictive performance. Together, these directions focus on expanding transport coverage, improving uncertainty and calibration, preserving quantitative permeability information, and strengthening prospective validation.

## 7. Conclusions

MechBBB is a two-stage BBB permeability classifier that combines physicochemical descriptors, ECFP4 fingerprints, and Stage 1 scores from efflux, influx, and PAMPA models. Model C achieved raw AUROC of 0.932 and AUPRC of 0.983 on the BBBP scaffold test set, with MCC of 0.737 and balanced accuracy of 0.866 at the validation-selected threshold of 0.51. On the strict B3DB external set of 4080 compounds, Model C achieved raw AUROC of 0.894 and AUPRC of 0.893, with MCC of 0.634 and balanced accuracy of 0.808, without retraining, recalibration, or threshold adjustment. The improvement over Model B was modest on BBBP, and no statistically significant external ranking advantage was established. These results support strong absolute performance while limiting the claim of incremental benefit to the evidence observed.

The corrected SHAP analysis showed that TPSA, NumHDonors, and p_pampa were among the leading contributors, with p_efflux and p_influx also contributing to the fitted classifier. These findings indicate that the model uses both physicochemical information and learned transport-related scores, but they do not establish biological causality or identify the transporter responsible for an individual prediction [41]. The pooled SLC labels and absence of separate BCRP and MRP models limit the mechanistic specificity of the current approach. In addition, calibration improved on the BBBP test set but deteriorated on strict B3DB, indicating that predicted probabilities should be interpreted cautiously under external distribution shift.

Taken together, MechBBB provides a leakage-controlled framework for BBB permeability screening that combines competitive prediction with transport-related information for individual compounds. On the matched strict B3DB benchmark, MechBBB achieved higher accuracy, balanced accuracy, sensitivity, and MCC than SwissADME BOILED-Egg, although BOILED-Egg showed higher specificity. The Stage 1 scores did not produce a consistent ranking advantage over the descriptor-plus-fingerprint baseline across all evaluations, but they provide additional information on predicted efflux, influx, and passive-permeability behavior. This information can help prioritize compounds for experimental BBB testing and identify candidates that warrant follow-up transporter or permeability studies during CNS drug discovery. MechBBB should therefore be viewed as a screening and prioritization tool rather than a replacement for experimental BBB measurements. Future work should expand transporter-specific coverage, incorporate continuous permeability endpoints, and evaluate uncertainty and calibration on prospective datasets. Source code, trained model files, and the web interface are freely available at https://github.com/sivaGU/MechBBB (accessed on 22 September 2026) and https://mechbbb.streamlit.app/. (accessed on 22 September 2026).

## Figures and Tables

**Figure 1:**
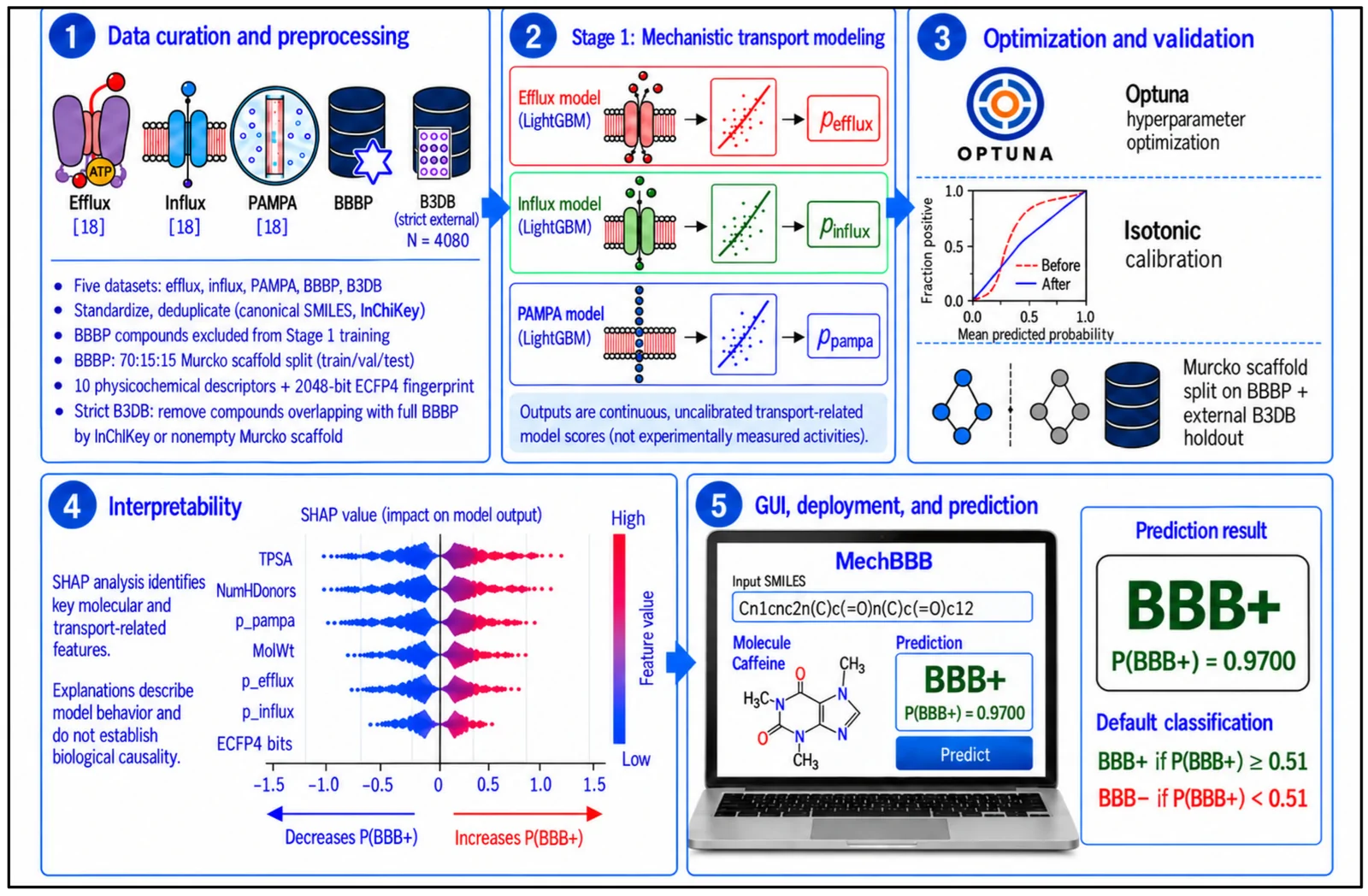
Overview of the MechBBB workflow. (**1**) Data curation and preprocessing of the efflux, influx, PAMPA, BBBP, and B3DB datasets [18], including molecular standardization, duplicate removal, and leakage control [27]. (**2**) Stage 1 LightGBM models generate continuous efflux, influx, and passive permeability scores [23]. (**3**) Stage 2 optimization, scaffold-based validation, and isotonic calibration are performed using BBBP, followed by evaluation on the held-out BBBP test set and strict B3DB external set [28,29]. (**4**) SHAP analysis is used to describe contributions from physicochemical descriptors, ECFP4 fingerprints, and Stage 1 scores [26]. (**5**) The final frozen model is deployed through the MechBBB graphical user interface for single-molecule and batch prediction (Figure Created in BioRender).

**Figure 2:**
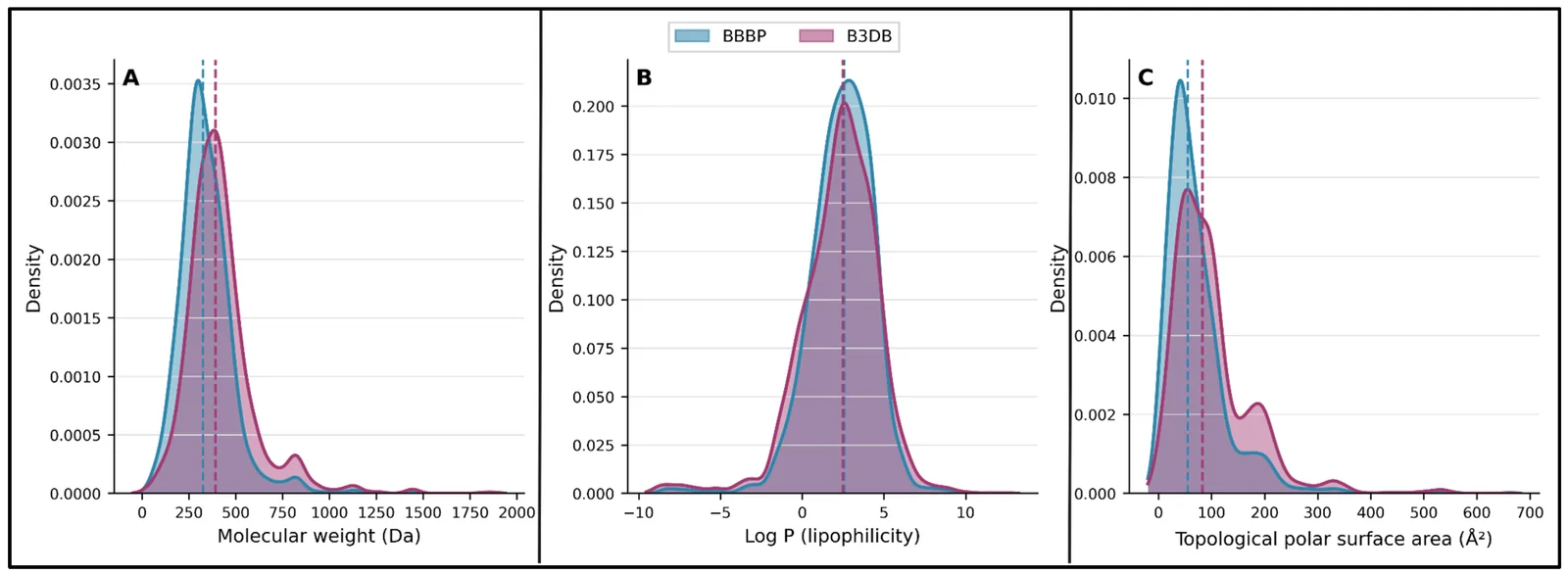
Physicochemical property distributions for the BBBP training set (*n* = 1365) and the strict B3DB external test set (*n* = 4080). (**A**) Molecular weight (MW). (**B**) Log P (lipophilicity). (**C**) Topological polar surface area (TPSA). The strict B3DB set was built by removing compounds that overlap the full BBBP dataset by InChIKey or nonempty Murcko scaffold. The dashed lines mark the median of each set. The plots show differences in physicochemical properties between the two populations. Only the BBBP training set was used for model fitting, and B3DB was kept for external testing.

**Figure 3:**
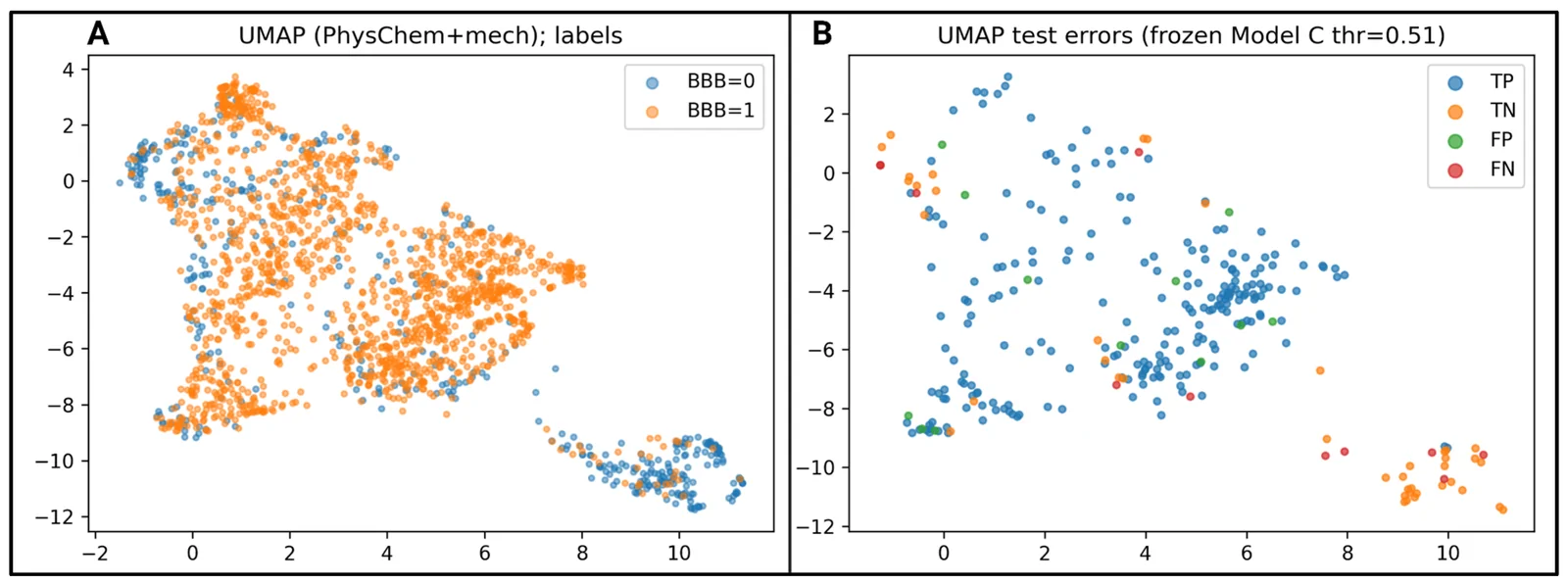
UMAP view of BBBP chemical space and Model C test set outcomes. (**A**) UMAP of the full BBBP dataset (*n* = 1950) colored by BBB label, where BBB− and BBB+ mark the two classes. (**B**) The held-out BBBP test compounds (*n* = 293) were placed in the same UMAP space and colored by the outcome from the frozen Model C, true positive (TP), true negative (TN), false positive (FP), and false negative (FN). Outcomes were set from the P(BBB+) values of Model C and the fixed threshold of 0.51. The UMAP came from the physicochemical and Stage 1 score inputs and was used for viewing only [36]. The placed coordinates carry no direct physicochemical reading and do not set biological mechanism.

**Figure 4:**
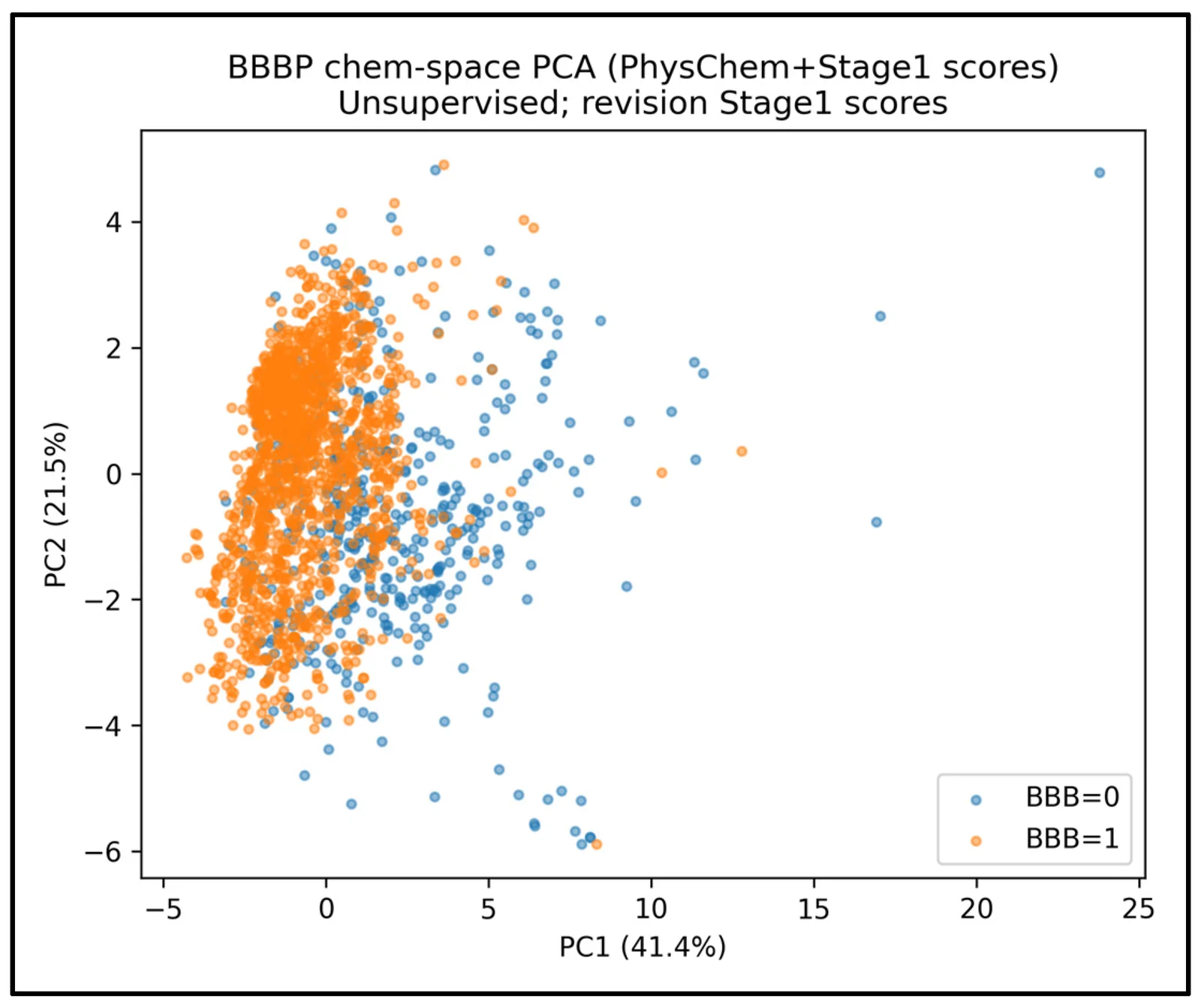
PCA of the BBBP continuous input space. PCA was run on 13 inputs made of 10 physicochemical descriptors and three Stage 1 transport scores, p_efflux, p_influx, and p_pampa [37]. The 2048-bit ECFP4 fingerprint was not part of this PCA. Points show BBBP compounds colored by BBB label. PC1 held 41.4% of the total variance and PC2 held 21.5% for total of 62.9% over the two shown components. The PCA was used for viewing only and was not an input-reduction step in Stage 2 training.

**Figure 5:**
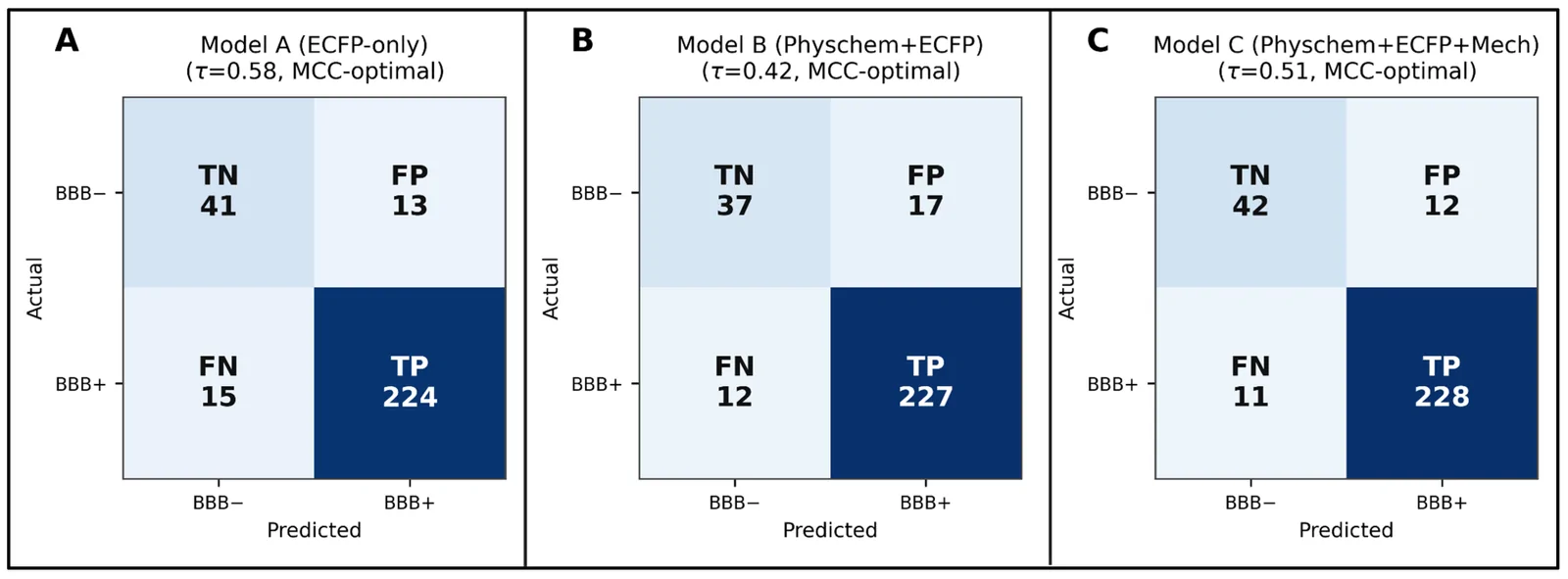
Confusion matrices for Model A, Model B, and Model C on the BBBP test set (*n* = 293) at their MCC optimal thresholds. (**A**) Model A (ECFP only) at threshold 0.58. (**B**) Model B (physicochemical descriptors plus ECFP) at threshold 0.42. (**C**) Model C (physicochemical descriptors plus ECFP plus Stage 1 scores) at threshold 0.51. Each panel reports true negative, false positive, false negative, and true positive counts at the validation selected threshold. Darker shading indicates a higher count.

**Figure 6:**
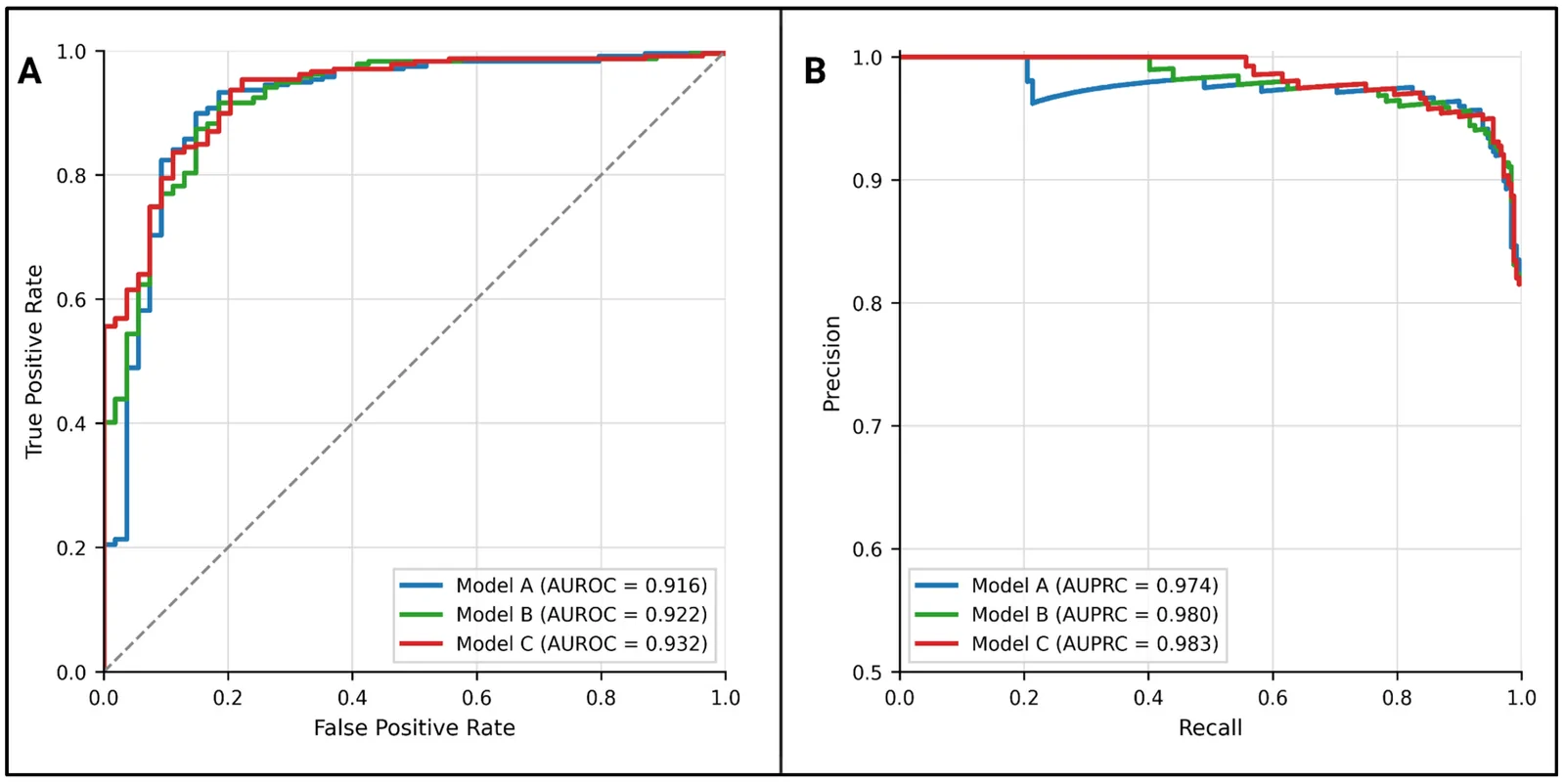
ROC and precision recall curves for Model A, Model B, and Model C on the BBBP scaffold split test set (*n* = 293). (**A**) ROC curves with AUROC in the legend. Model A uses ECFP4 fingerprints only. Model B uses ten physicochemical descriptors plus ECFP4 fingerprints. Model C uses ten physicochemical descriptors plus ECFP4 fingerprints plus three Stage 1 scores. (**B**) Precision recall curves for the same models. Model C scores come from a five seed average, with the per-molecule scores averaged over the seeds before reporting. The curves show ranking performance over all thresholds and do not depend on one decision threshold [33,34].

**Figure 7:**
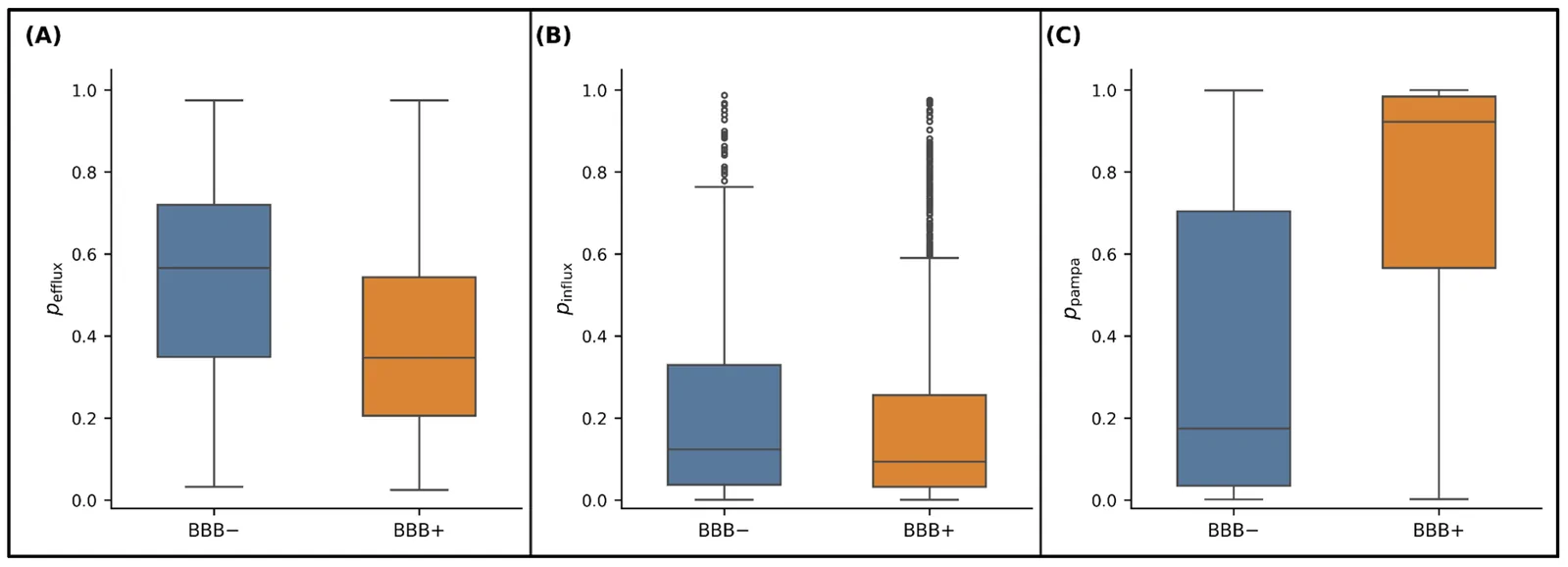
Distributions of the corrected Stage 1 scores on the cleaned BBBP dataset by experimental BBB label (*n* = 1950, BBB+ *n* = 1491, BBB− *n* = 459). Boxplots show the model scores for (**A**) p_efflux, (**B**) p_influx, and (**C**) p_pampa. Each box shows the median and the interquartile range. Whiskers reach 1.5 times the interquartile range, and points past the whiskers mark single compounds. The scores came from the corrected Stage 1 refit models and are not direct transporter or PAMPA measurements.

**Figure 8:**
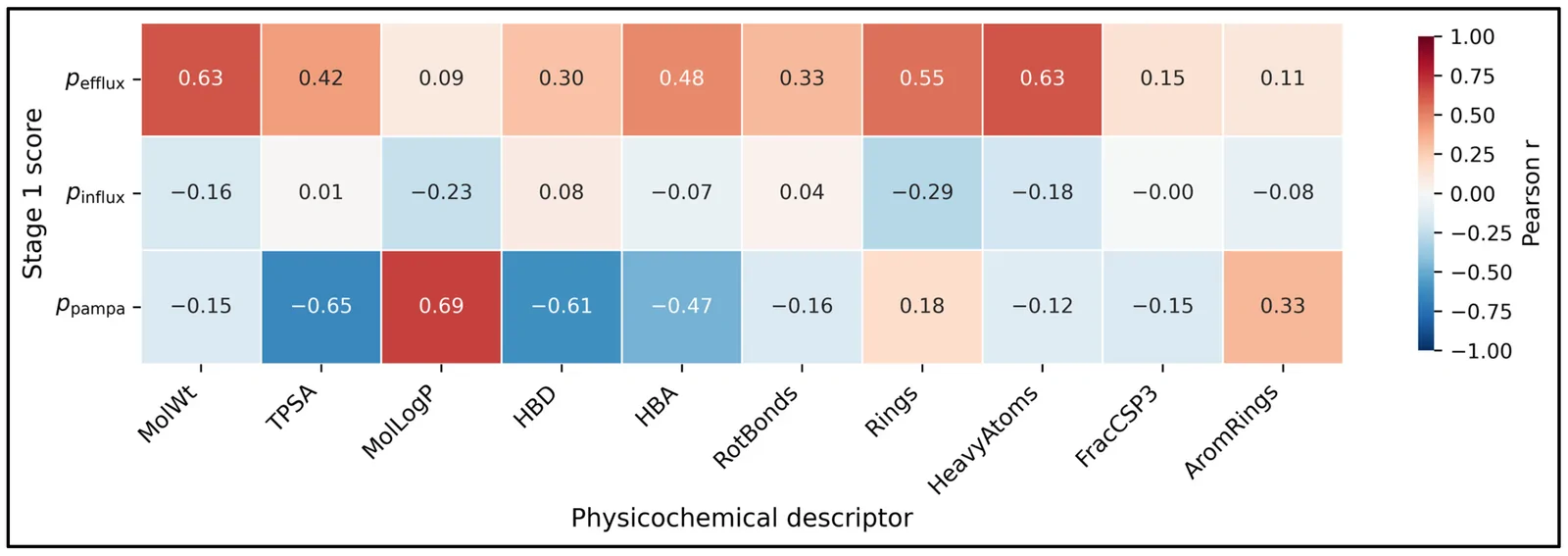
Pearson correlation heatmap between the corrected Stage 1 scores and the 10 physicochemical descriptors used in Stage 2 on the cleaned BBBP dataset (*n* = 1950). Correlations are shown for p_efflux, p_influx, and p_pampa against MolWt, TPSA, MolLogP, NumHDonors, NumHAcceptors, NumRotatableBonds, RingCount, HeavyAtomCount, FractionCSP3, and NumAromaticRings. The strongest links were p_pampa with MolLogP (r = +0.687) and p_efflux with MolWt (r = +0.630). p_influx showed weaker links with the descriptors, and its largest absolute value was with RingCount (r = −0.287). All correlations came from the corrected refit scores paired with the Stage 2 descriptor table.

**Figure 9:**
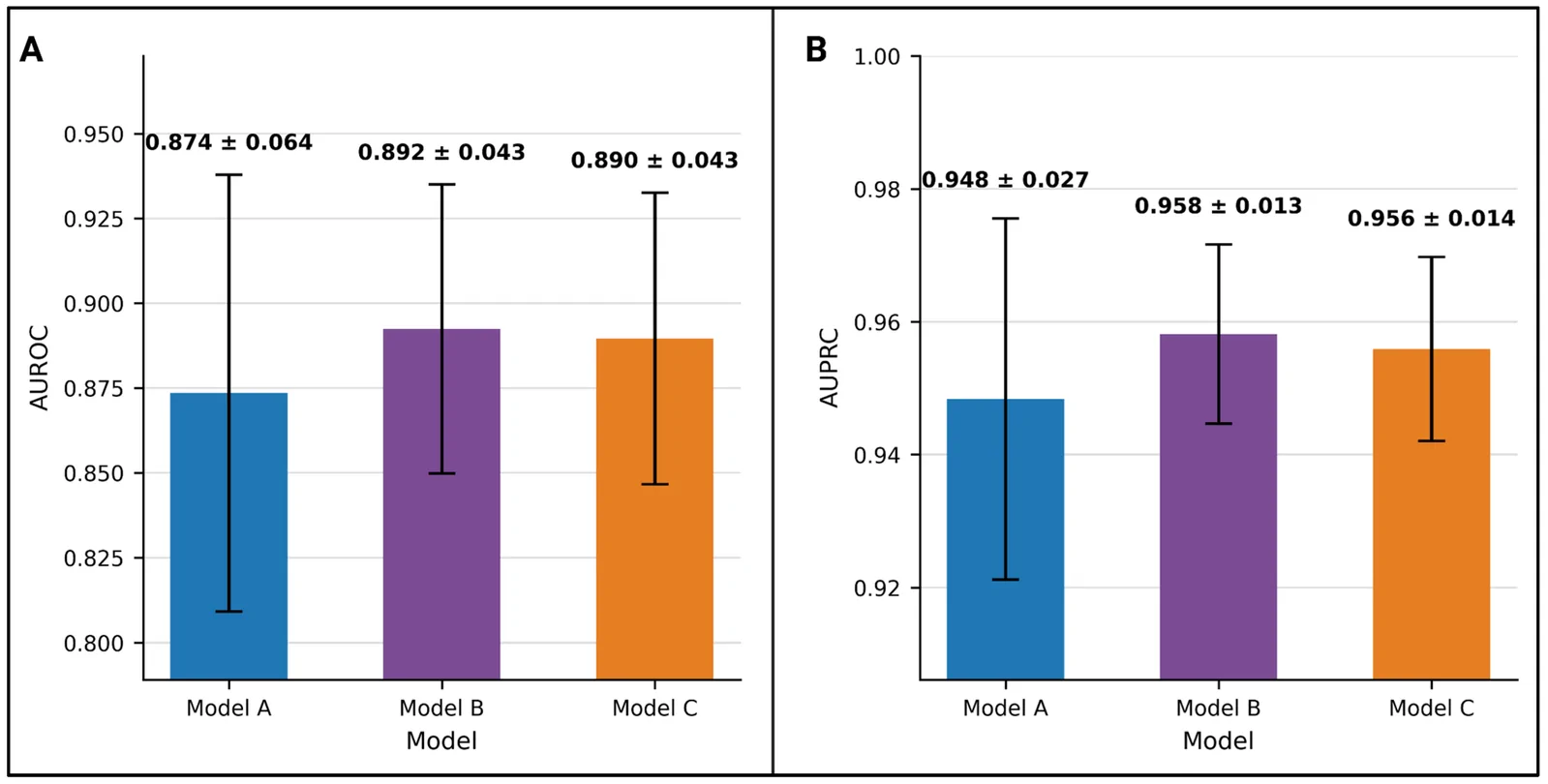
Five-fold scaffold-grouped cross-validation results for Models A, B, and C on the BBBP training and validation set. (**A**) AUROC and (**B**) AUPRC averaged across five Murcko scaffold-grouped folds. Bars show the mean across folds, and error bars show one standard deviation. Model A uses ECFP4 only. Model B uses physicochemical descriptors and ECFP4. Model C uses physicochemical descriptors, ECFP4, and Stage 1 scores (p_efflux, p_influx, p_pampa).

**Figure 10:**
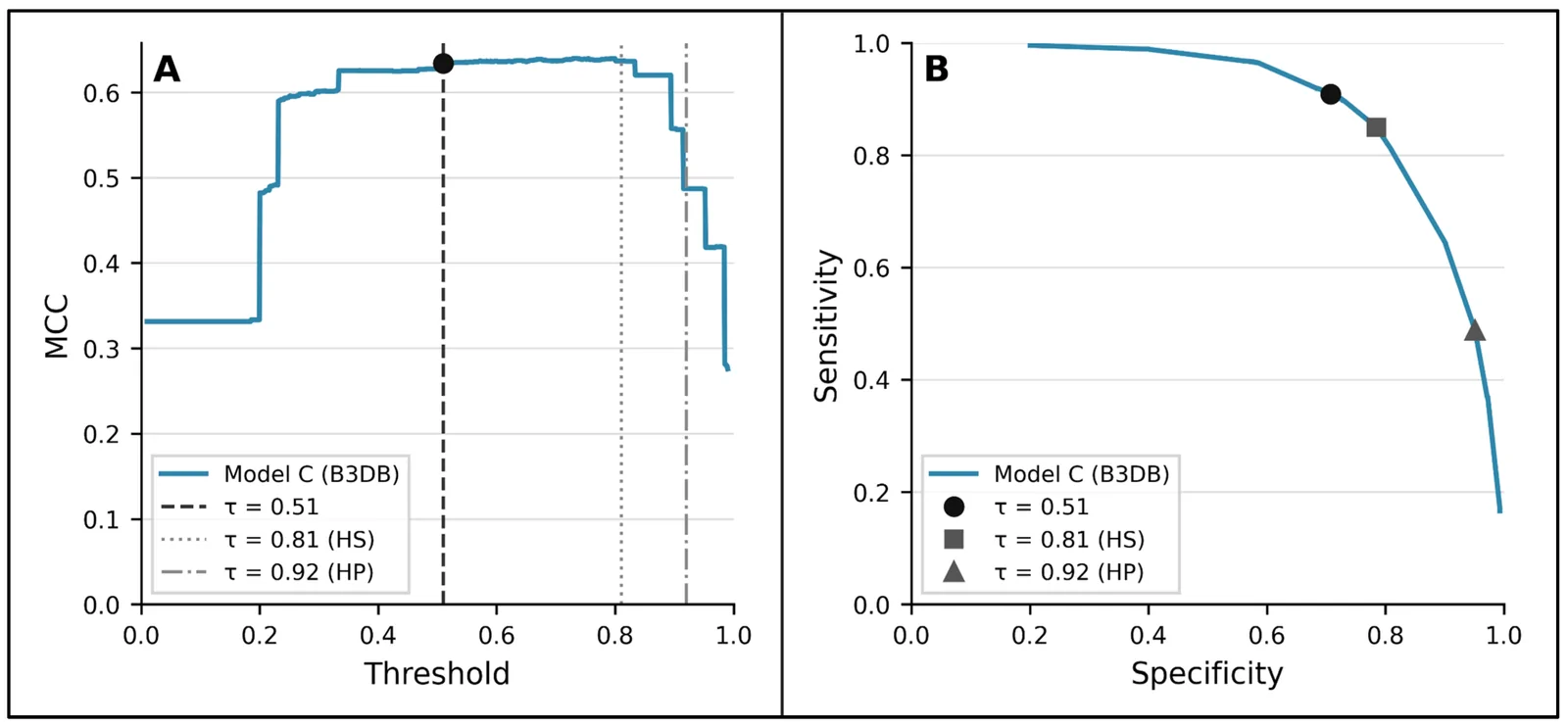
Threshold robustness analysis for Model C on the strict B3DB external set (*n* = 4080, 53.2% BBB+). (**A**) MCC against decision threshold, with the fixed primary threshold τ = 0.51 and the two-validation set operating points τ = 0.81 and τ = 0.92 marked. (**B**) Sensitivity and specificity trade-off curve, with the same three operating points marked. All thresholds were set on BBBP validation and applied with no change to B3DB. The threshold sweep is descriptive and was not used to set a new external operating point.

**Figure 11:**
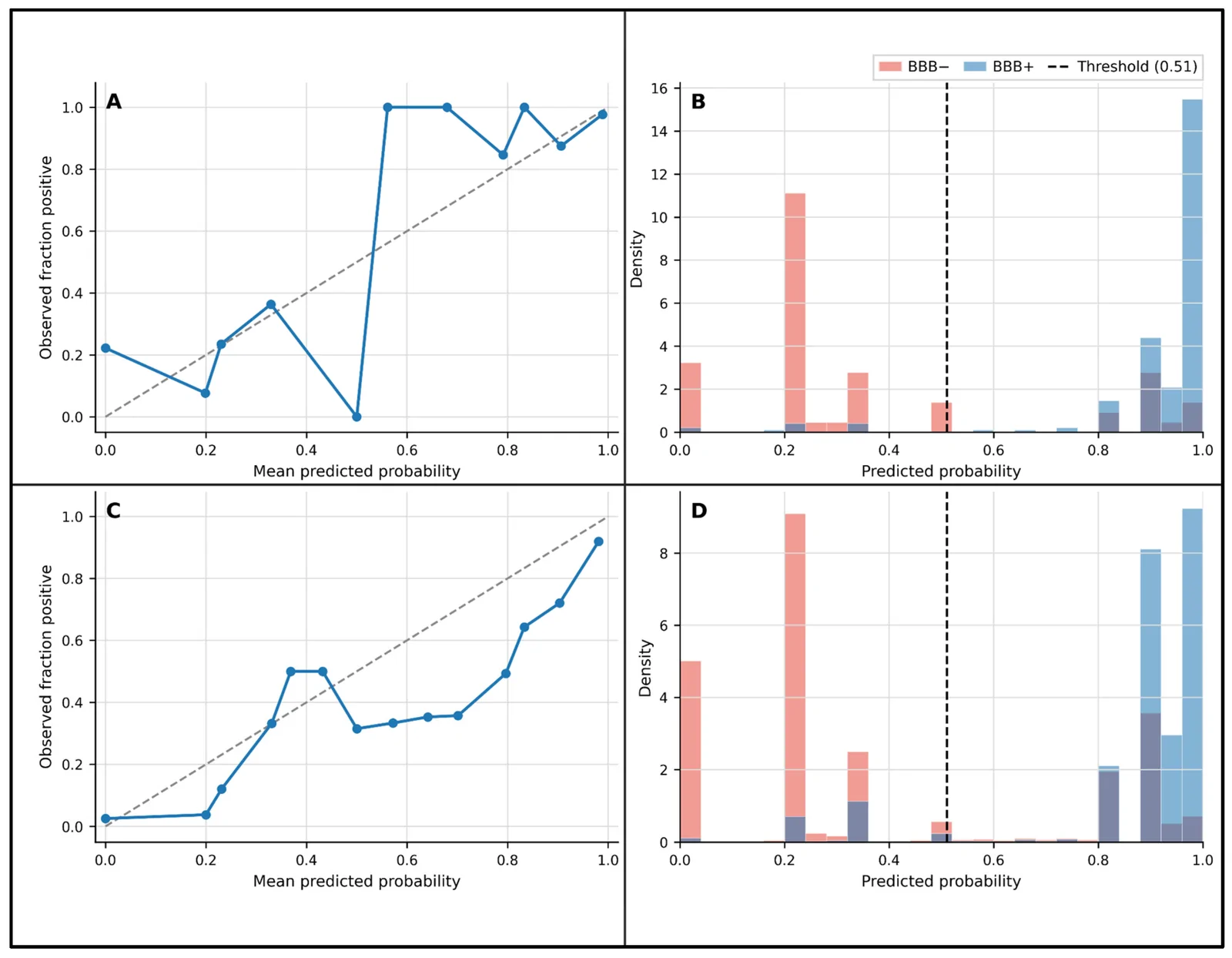
Probability calibration and score distributions for MechBBB (Model C) on the held-out BBBP test set and strict B3DB [40]. (**A**) Reliability diagram for the BBBP test set (*n* = 293), comparing raw and calibrated predicted probabilities with observed BBB+ fractions. (**B**) Distribution of calibrated probabilities for BBB+ and BBB− compounds on the BBBP test set, with the fixed primary threshold of 0.51 marked. (**C**) Reliability diagram for strict B3DB (*n* = 4080), using the isotonic mapping fitted on BBBP validation without refitting. (**D**) Distribution of calibrated probabilities for BBB+ and BBB− compounds on strict B3DB, with the same fixed threshold marked. For Model C, BBBP test ECE changed from 0.0720 to 0.0482 and Brier score from 0.0822 to 0.0710 after calibration. On strict B3DB, ECE changed from 0.0488 to 0.1201 and Brier score from 0.1312 to 0.1495. The reliability diagrams describe calibration on the evaluated populations and do not establish causal explanations for differences between datasets. The diagonal dashed line indicates perfect calibration.

**Figure 12:**
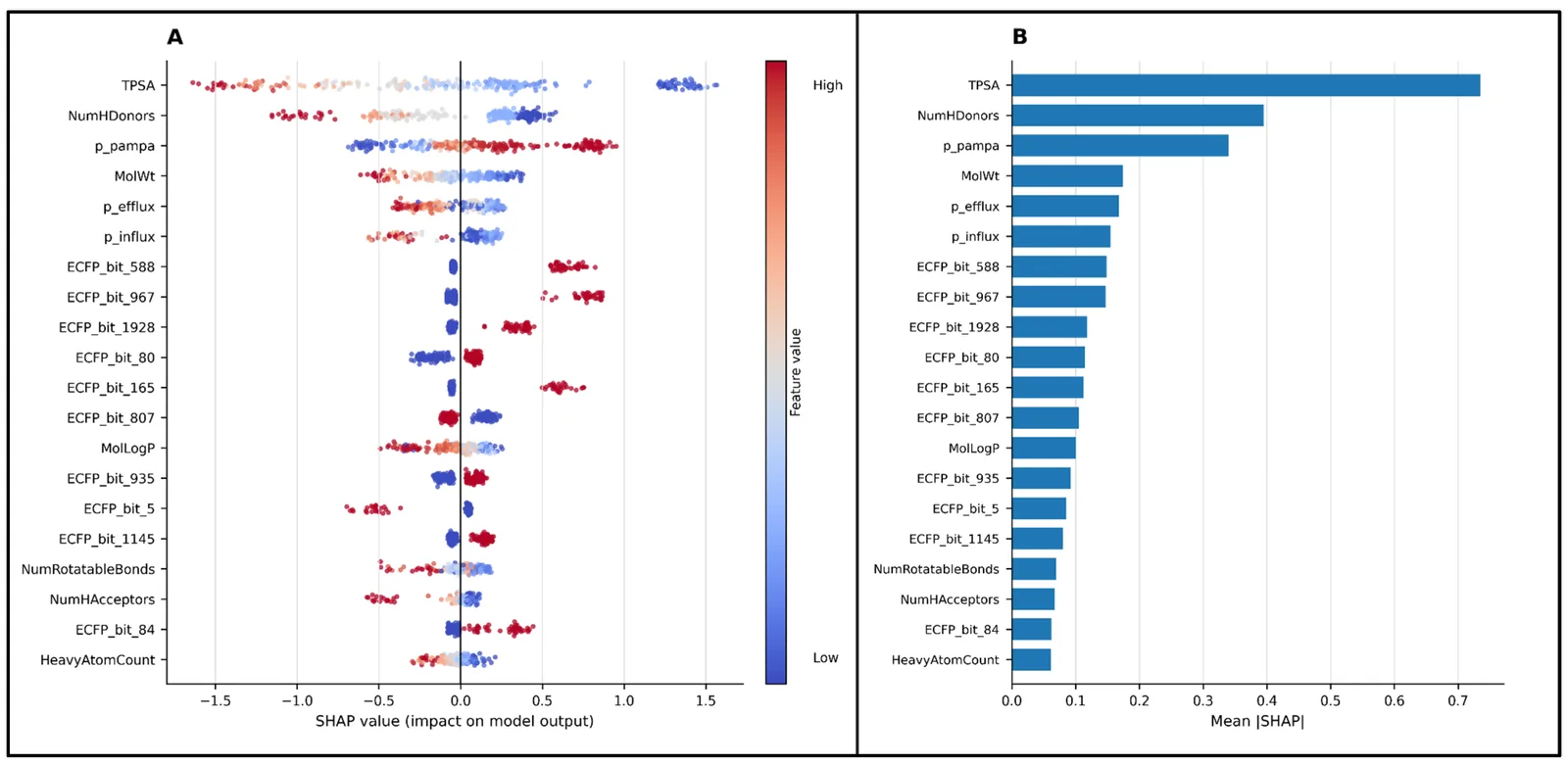
Global SHAP analysis of Model C on the held out BBBP test set [26]. (**A**) Beeswarm plot showing feature contributions across compounds. Color indicates feature value from low to high, and horizontal position indicates the SHAP contribution on the raw model margin scale. (**B**) Feature importance ranked by mean absolute SHAP value across test compounds. SHAP values were calculated for each of the five frozen LightGBM models and averaged across models before summarization. The plots describe model attribution rather than biological causality or additive contributions to the final isotonic calibrated probability.

**Figure 13:**
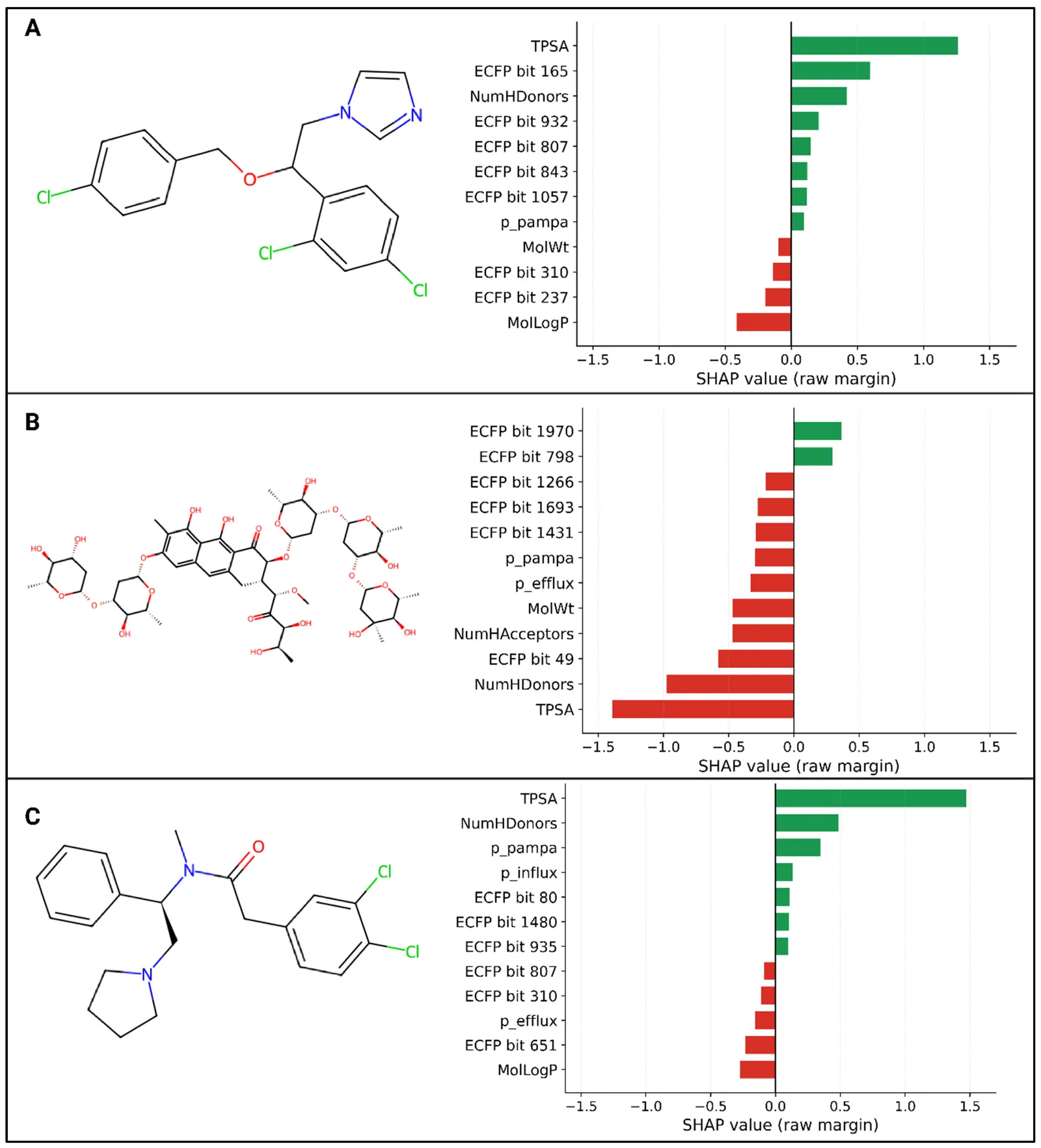
Individual compound SHAP explanations for three representative compounds from the held-out BBBP test set. (**A**) False-positive compound with experimental label BBB− and predicted label BBB+ (calibrated P(BBB+) = 0.984). (**B**) False negative compound with experimental label BBB+ and predicted label BBB− (calibrated P(BBB+) = 0.000). (**C**) Correctly classified true positive compound with experimental and predicted labels BBB+ (calibrated P(BBB+) = 0.984). Molecular structures are shown on the left, and the largest positive and negative SHAP contributions are shown on the right. Green bars indicate contributions that increase the raw Model C margin toward BBB+, whereas red bars indicate contributions that decrease the raw margin. SHAP values were calculated for each frozen ensemble member and averaged across the five models [26]. These values explain the raw model margin rather than the isotonic calibrated probability.

**Figure 14:**
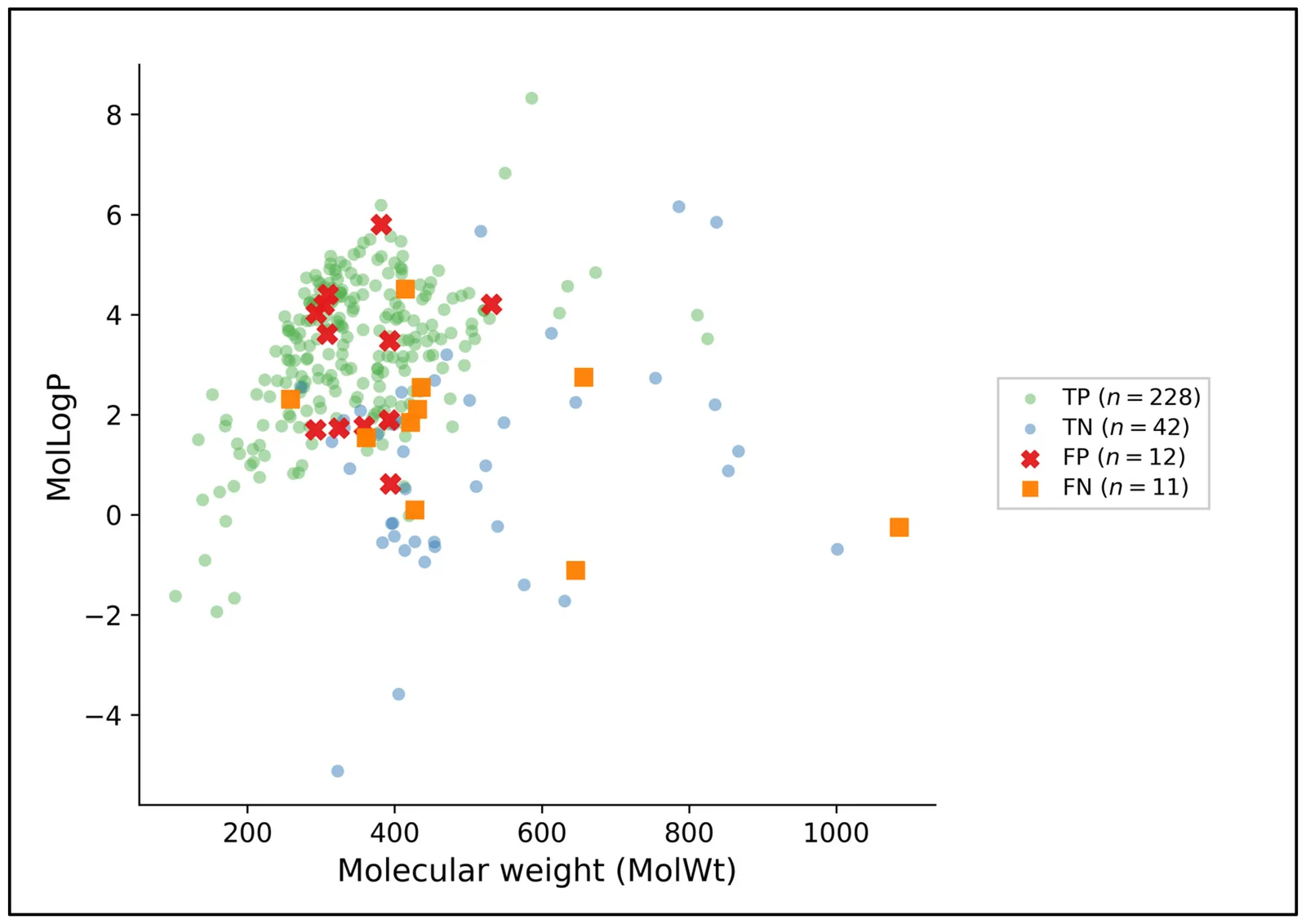
Error localization for Model C on the held-out BBBP test set (*n* = 293) in the molecular weight and MolLogP plane. True positives (TP, *n* = 228), true negatives (TN, *n* = 42), false positives (FP, *n* = 12), and false negatives (FN, *n* = 11) are shown using distinct markers. Classification outcomes were determined from calibrated probabilities using the fixed validation-selected threshold of 0.51. Molecular weight and MolLogP are shown only as descriptive reference properties. Proximity between compounds in this two-dimensional plot does not establish structural similarity or the biological cause of an error.

**Figure 15:**
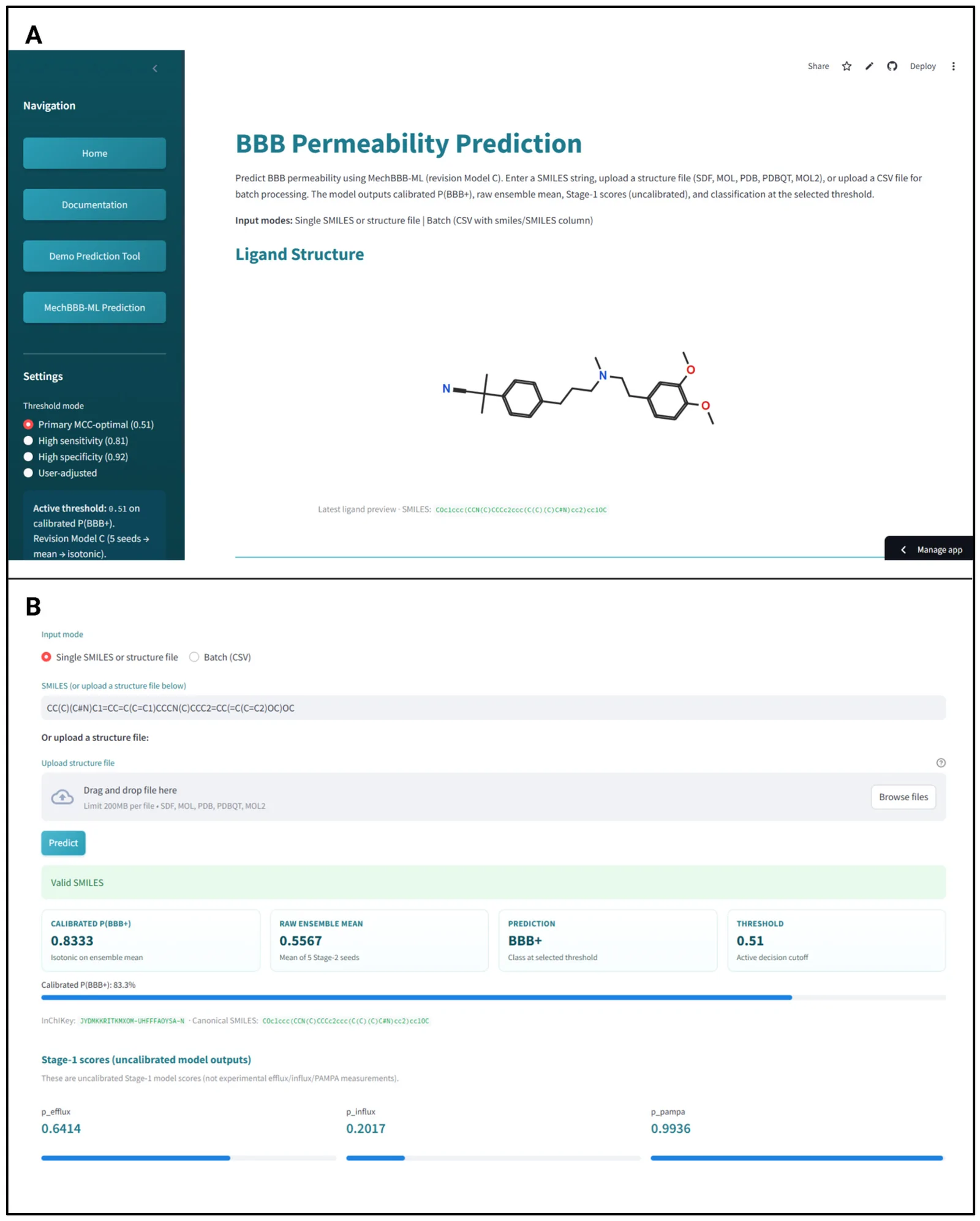
MechBBB interface illustrated with verapamil. (**A**) Home page showing the molecular structure, canonical SMILES, navigation, and threshold controls. (**B**) Prediction page showing the calibrated P(BBB+), raw ensemble mean, classification at the selected threshold, and the three uncalibrated Stage 1 scores. The interface supports single molecule and batch prediction.

**Table 1:** Dataset sizes and class balance. MoleculeNet BBBP was divided into training, validation, and test partitions using Murcko scaffold grouping. The strict B3DB external set was constructed by removing InChIKey and nonempty Murcko scaffold overlap against all BBBP partitions. BBB+ fraction denotes the proportion of compounds labeled BBB+.

Dataset	Partition	N	BBB+	BBB−	BBB+ Fraction
BBBP	Training	1365	1014	351	0.743
BBBP	Validation	292	238	54	0.815
BBBP	Test	293	239	54	0.816
B3DB	Before overlap filtering	7804	4955	2849	0.635
B3DB	Strict external set	4080	2170	1910	0.532

**Table 2:** Model performance on the held out BBBP scaffold test set (*n* = 293). Model A uses ECFP4 fingerprints, Model B adds ten physicochemical descriptors, and Model C adds the three Stage 1 scores to Model B. AUROC and AUPRC were calculated from raw ensemble mean scores. MCC and balanced accuracy were calculated from calibrated probabilities at each model’s validation selected threshold. Values in brackets are 95% class stratified bootstrap confidence intervals [38]. The bootstrap procedure is described in Section 3.8.1. Complete BBBP test metrics are provided in Supplementary Table S9.

Measure	Model A	Model B	Model C
Threshold	0.58	0.42	0.51
AUROC	0.916 [0.862–0.959]	0.922 [0.879–0.959]	0.932 [0.895–0.963]
AUPRC	0.974 [0.954–0.990]	0.980 [0.967–0.990]	0.983 [0.974–0.991]
MCC	0.687 [0.579–0.793]	0.660 [0.539–0.767]	0.737 [0.632–0.834]
Balanced accuracy	0.848 [0.786–0.905]	0.817 [0.751–0.878]	0.866 [0.805–0.921]

**Table 3:** Model C performance at three fixed operating points on the held-out BBBP scaffold test set. Thresholds were selected using calibrated probabilities on the BBBP validation set. The primary threshold maximized MCC. The high sensitivity threshold maximized specificity subject to validation sensitivity ≥ 0.90, and the high specificity threshold maximized sensitivity subject to validation specificity ≥ 0.90. Thresholds were applied unchanged to the test set. The reported sensitivity, specificity, and MCC are test set results.

Operating Point	Threshold	Sensitivity	Specificity	MCC
MCC optimal	0.51	0.954	0.778	0.737
High Sensitivity	0.81	0.900	0.815	0.656
High Specificity	0.92	0.703	0.926	0.495

**Table 4:** Stage 1 performance under fair random and Murcko scaffold-based testing. Both evaluations used the corrected leak safe datasets and matched training procedures. Δ values were calculated as scaffold minus random performance. The holdouts contained different compounds, so no paired statistical test was performed. AUPRC differences should be interpreted alongside positive class prevalence, particularly for the influx task [32].

Stage 1 Task	N	Metric	Random	Scaffold	Δ
Efflux	2236	AUROC	0.8182	0.7639	−0.0543
Efflux	2236	AUPRC	0.8743	0.8284	−0.0459
Influx	807	AUROC	0.9463	0.9369	−0.0094
Influx	807	AUPRC	0.8539	0.9079	+0.0540
PAMPA	1442	AUROC	0.8998	0.9006	+0.0008
PAMPA	1442	AUPRC	0.9697	0.9665	−0.0032

**Table 5:** Five-fold Murcko scaffold-grouped cross-validation on the combined BBBP training and validation partitions (*n* = 1657). Values are mean ± standard deviation across folds. Model A uses ECFP4 fingerprints, Model B adds ten physicochemical descriptors, and Model C adds the three Stage 1 scores to Model B. The held out BBBP test set was excluded. This analysis assessed ranking stability using locked hyperparameters and was not used for further model selection.

Model	AUROC	AUPRC
Model A	0.874 ± 0.064	0.948 ± 0.027
Model B	0.892 ± 0.043	0.958 ± 0.013
Model C	0.890 ± 0.043	0.956 ± 0.014

**Table 6:** External validation results on the strict B3DB set (*n* = 4080) after InChIKey and nonempty Murcko scaffold overlap removal against the full BBBP dataset. Model predictions were made without retraining on B3DB. AUROC and AUPRC were computed from raw five seed mean scores before isotonic adjustment. MCC and balanced accuracy were computed from adjusted scores at the fixed validation set thresholds. Bootstrap 95% confidence intervals are shown in brackets.

Model	Threshold	AUROC	AUPRC	MCC	Balanced Accuracy
Model A	0.58	0.883 [0.873–0.893]	0.887 [0.876–0.897]	0.595 [0.571–0.620]	0.794 [0.782–0.807]
Model B	0.42	0.895 [0.885–0.904]	0.894 [0.882–0.905]	0.630 [0.608–0.652]	0.803 [0.792–0.815]
Model C	0.51	0.894 [0.884–0.904]	0.893 [0.881–0.904]	0.634 [0.611–0.656]	0.808 [0.797–0.820]

**Table 7:** Physicochemical properties and Stage 1 scores by Model C prediction outcome on the held out BBBP test set at threshold 0.51. Except for N, values are median (interquartile range). MolWt is expressed in Da and TPSA in Å^2^. All listed variables were available for every compound. TP, true positive; TN, true negative; FP, false positive; FN, false negative. Stage 1 scores are learned transport-related outputs rather than experimental measurements.

Variable	TP	TN	FP	FN
N	228	42	12	11
MolWt	335.01 (129.09)	446.98 (171.16)	341.42 (85.47)	427.46 (152.83)
MolLogP	3.513 (1.855)	1.268 (2.746)	3.545 (2.440)	1.847 (1.608)
TPSA	47.06 (48.64)	156.26 (80.30)	68.29 (39.29)	112.74 (68.96)
p_efflux	0.384 (0.394)	0.614 (0.278)	0.286 (0.344)	0.746 (0.221)
p_influx	0.080 (0.133)	0.151 (0.287)	0.037 (0.120)	0.113 (0.500)
p_pampa	0.944 (0.277)	0.097 (0.334)	0.538 (0.841)	0.202 (0.377)

**Table 8:** Blood–brain barrier classification performance of MechBBB Model C and published models under scaffold-based evaluation. Model C values are from the present study, covering the held out BBBP scaffold test set (*n* = 293) and the strict external B3DB set (*n* = 4080). The three published BBBP scaffold AUROC values are reported in Table 1 of Qin et al. [52]. References [15,53] identify the original FP-GNN and Chemprop methods. Published studies used an 8:1:1 scaffold split, whereas the present study used 70:15:15. A dash indicates a metric not reported in the cited source. Cross-study values provide context and do not establish a direct ranking.

Method	Reference	Dataset	Split	AUROC	Accuracy	F1
Model C (ours)	This work	BBBP	Scaffold 70:15:15	0.932	0.922	0.952
Model C (ours)	This work	B3DB external	External overlap controlled, *n* = 4080	0.894	0.815	0.839
MoleculeFormer	[52]	BBBP	Scaffold 8:1:1	0.924	-	-
FP-GNN	[52,53]	BBBP	Scaffold 8:1:1	0.916	-	-
Chemprop (optimized)	[15,52]	BBBP	Scaffold 8:1:1	0.886	-	-

**Table 9:** Head-to-head comparison of MechBBB Model C and the official SwissADME BOILED-Egg method on the chemically verified matched strict B3DB subset (*n* = 4043; BBB+ *n* = 2166; BBB− *n* = 1877) [54,55]. Model C used the frozen calibrated predictions and validation selected threshold of 0.51. BOILED-Egg was evaluated using its official exported binary BBB classification. AUROC and AUPRC are not reported for BOILED-Egg because the exported output is binary rather than a continuous prediction score.

Method	N	Accuracy	Balanced Accuracy	Sensitivity	Specificity	MCC
MechBBB	4043	0.814	0.806	0.910	0.703	0.631
SwissADME BOILED-Egg	4043	0.682	0.693	0.541	0.845	0.401

## Data Availability

The original data presented in the study are openly available in the MechBBB GitHub repository at https://github.com/sivaGU/MechBBB (accessed on 22 September 2026). The BBBP, B3DB, and transport datasets were obtained from the publicly available sources cited in the manuscript and remain subject to their respective terms. The trained prediction workflow is accessible at https://mechbbb.streamlit.app/ (accessed on 22 September 2026). Additional intermediate computational files that are not included in the repository will be made available by the authors on request.

## Supplementary Materials

The following supporting information can be downloaded at: https://www.mdpi.com/article/10.3390/ph19101524/s1, Table S1: Datasets and data splits; Table S2: Compound level dataset inventory and curation records; Table S3: Feature schema and input definitions; Table S4: Stage 1 fitting protocol and model parameters; Table S5: Stage 2 hyperparameter optimization; Table S6: Calibration and threshold selection; Table S7: B3DB external validation and statistical protocol; Table S8: Compound level predictions; Table S9: BBBP test metrics; Table S10: Paired statistical comparisons and robustness analyses; Table S11: SwissADME BOILED-Egg benchmark; Table S12: Interpretability supporting data; Table S13: Stage 1 random and scaffold based evaluation; Table S14: Stage 1 score behavior and descriptor correlations; Table S15: Stage 2 scaffold grouped cross validation; Table S16: Strict B3DB external validation metrics. Supplementary Codes S1–S27 provide the scripts used for dataset ingestion, chemical standardization, scaffold splitting, feature generation, Stage 1 modeling, Stage 2 model development, calibration, locked evaluation, statistical analysis, SwissADME benchmarking, interpretability analysis, and figure generation.

## References

[1] Pardridge W.M. (2012). Drug Transport across the Blood–Brain Barrier. J. Cereb. Blood Flow Metab..

[2] Abbott N.J., Patabendige A.A.K., Dolman D.E.M., Yusof S.R., Begley D.J. (2010). Structure and function of the blood-brain barrier. Neurobiol. Dis..

[3] Daneman R., Prat A. (2015). The Blood–Brain Barrier. Cold Spring Harb. Perspect. Biol..

[4] Miller D.S. (2010). Regulation of P-glycoprotein and other ABC drug transporters at the blood–brain barrier. Trends Pharmacol. Sci..

[5] Strazielle N., Ghersi-Egea J.-F. (2015). Efflux transporters in blood-brain interfaces of the developing brain. Front. Neurosci..

[6] Lin L., Yee S.W., Kim R.B., Giacomini K.M. (2015). SLC transporters as therapeutic targets: Emerging opportunities. Nat. Rev. Drug Discov..

[7] Sugano K., Kansy M., Artursson P., Avdeef A., Bendels S., Di L., Ecker G.F., Faller B., Fischer H., Gerebtzoff G. (2010). Coexistence of passive and carrier-mediated processes in drug transport. Nat. Rev. Drug Discov..

[8] Schinkel A., Smit J., van Tellingen O., Beijnen J., Wagenaar E., van Deemter L., Mol C., van der Valk M., Robanus-Maandag E., Riele H.T. (1994). Disruption of the mouse mdr1a P-glycoprotein gene leads to a deficiency in the blood-brain barrier and to increased sensitivity to drugs. Cell.

[9] Giacomini K.M., Huang S.M., Tweedie D.J., Benet L.Z., Brouwer K.L.R., Chu X., Dahlin A., Evers R., The International Transporter Consortium, International Transporter Consortium (2010). Membrane transporters in drug development. Nat. Rev. Drug Discov..

[10] Hitchcock S.A., Pennington L.D. (2006). Structure−Brain Exposure Relationships. J. Med. Chem..

[11] Rankovic Z. (2015). CNS Drug Design: Balancing Physicochemical Properties for Optimal Brain Exposure. J. Med. Chem..

[12] Wager T.T., Hou X., Verhoest P.R., Villalobos A. (2010). Moving beyond Rules: The Development of a Central Nervous System Multiparameter Optimization (CNS MPO) Approach to Enable Alignment of Druglike Properties. ACS Chem. Neurosci..

[13] Martins I.F., Teixeira A.L., Pinheiro L., Falcao A.O. (2012). A Bayesian Approach to in Silico Blood-Brain Barrier Penetration Modeling. J. Chem. Inf. Model..

[14] Shaker B., Yu M.-S., Song J.S., Ahn S., Ryu J.Y., Oh K.-S., Na D. (2020). LightBBB: Computational prediction model of blood–brain-barrier penetration based on LightGBM. Bioinformatics.

[15] Yang K., Swanson K., Jin W., Coley C., Eiden P., Gao H., Guzman-Perez A., Hopper T., Kelley B., Mathea M. (2019). Analyzing Learned Molecular Representations for Property Prediction. J. Chem. Inf. Model..

[16] Xiong Z., Wang D., Liu X., Zhong F., Wan X., Li X., Li Z., Luo X., Chen K., Jiang H. (2020). Pushing the Boundaries of Molecular Representation for Drug Discovery with the Graph Attention Mechanism. J. Med. Chem..

[17] Ross J., Belgodere B., Chenthamarakshan V., Padhi I., Mroueh Y., Das P. (2022). Large-scale chemical language representations capture molecular structure and properties. Nat. Mach. Intell..

[18] Cornelissen F.M., Markert G., Deutsch G., Antonara M., Faaij N., Bartelink I., Noske D., Vandertop W.P., Bender A., Westerman B.A. (2023). Explaining Blood–Brain Barrier Permeability of Small Molecules by Integrated Analysis of Different Transport Mechanisms. J. Med. Chem..

[19] Wu Z., Ramsundar B., Feinberg E.N., Gomes J., Geniesse C., Pappu A.S., Leswing K., Pande V. (2018). MoleculeNet: A benchmark for molecular machine learning. Chem. Sci..

[20] Meng F., Xi Y., Huang J., Ayers P.W. (2021). A curated diverse molecular database of blood-brain barrier permeability with chemical descriptors. Sci. Data.

[21] Kapoor S., Narayanan A. (2023). Leakage and the reproducibility crisis in machine-learning-based science. Patterns.

[22] Tropsha A. (2010). Best Practices for QSAR Model Development, Validation, and Exploitation. Mol. Inform..

[23] Ke G., Meng Q., Finley T., Wang T., Chen W., Ma W., Ye Q., Liu T.Y. (2017). LightGBM: A Highly Efficient Gradient Boosting Decision Tree. Adv. Neural Inf. Process. Syst..

[24] Rogers D., Hahn M. (2010). Extended-Connectivity Fingerprints. J. Chem. Inf. Model..

[25] Bemis G.W., Murcko M.A. (1996). The Properties of Known Drugs. 1. Molecular Frameworks. J. Med. Chem..

[26] Lundberg S.M., Erion G., Chen H., DeGrave A., Prutkin J.M., Nair B., Katz R., Himmelfarb J., Bansal N., Lee S.-I. (2020). From local explanations to global understanding with explainable AI for trees. Nat. Mach. Intell..

[27] Bento A.P., Hersey A., Félix E., Landrum G., Gaulton A., Atkinson F., Bellis L.J., De Veij M., Leach A.R. (2020). An open source chemical structure curation pipeline using RDKit. J. Cheminform..

[28] Akiba T., Sano S., Yanase T., Ohta T., Koyama M. (2019). Optuna: A Next-generation Hyperparameter Optimization Framework. Proceedings of the 25th ACM SIGKDD Interenational Conference on Knowledge Discovery & Data Mining.

[29] Niculescu-Mizil A., Caruana R. (2019). Predicting Good Probabilities with Supervised Learning. Proceedings of the 22nd International Conference on Machine Learning.

[30] Heller S.R., McNaught A., Pletnev I., Stein S., Tchekhovskoi D. (2015). InChI, the IUPAC International Chemical Identifier. J. Cheminform..

[31] Chicco D., Jurman G. (2020). The advantages of the Matthews correlation coefficient (MCC) over F1 score and accuracy in binary classification evaluation. BMC Genom..

[32] Saito T., Rehmsmeier M. (2015). The Precision-Recall Plot Is More Informative than the ROC Plot When Evaluating Binary Classifiers on Imbalanced Datasets. PLoS ONE.

[33] Davis J., Goadrich M. (2006). The Relationship Between Precision-Recall and ROC Curves. Proceedings of the 23rd International Conference on Machine Learning.

[34] Fawcett T. (2006). An introduction to ROC analysis. Pattern Recogn. Lett..

[35] Brodersen K.H., Ong C.S., Stephan K.E., Buhmann J.M. (2010). The Balanced Accuracy and Its Posterior Distribution. Proceedings of the 2010 20th International Conference on Pattern Recognition.

[36] McInnes L., Healy J., Saul N., Großberger L. (2018). UMAP: Uniform Manifold Approximation and Projection. J. Open Source Softw..

[37] Jolliffe I.T., Cadima J. (2016). Principal component analysis: A review and recent developments. Philos. Trans. R. Soc. A Math. Phys. Eng. Sci..

[38] Efron B. (1979). Bootstrap Methods: Another Look at the Jackknife. Ann. Stat..

[39] Delong E.R., Delong D.M., Clarke-Pearson D.L. (1988). Comparing the Areas under Two or More Correlated Receiver Operating Characteristic Curves: A Nonparametric Approach. Biometrics.

[40] van Calster B., McLernon D.J., van Smeden M., Wynants L., Steyerberg E.W., On behalf of Topic Group ‘Evaluating diagnostic tests and prediction models’ of the STRATOS initiative (2019). Calibration: The Achilles heel of predictive analytics. BMC Med..

[41] Kumar I.E., Venkatasubramanian S., Scheidegger C., Friedler S.A. (2020). Problems with Shapley-value-based explanations as feature importance measures. arXiv.

[42] Di L., Kerns E.H., Fan K., McConnell O.J., Carter G.T. (2003). High throughput artificial membrane permeability assay for blood–brain barrier. Eur. J. Med. Chem..

[43] Lakens D. (2013). Calculating and reporting effect sizes to facilitate cumulative science: A practical primer for t-tests and ANOVAs. Front. Psychol..

[44] Guo C., Pleiss G., Sun Y., Weinberger K.Q. (2017). On Calibration of Modern Neural Networks. arXiv.

[45] Brier G.W. (1950). Verification of Forecasts Expressed in Terms of Probability. Mon. Weather Rev..

[46] Rodríguez-Pérez R., Bajorath J. (2020). Interpretation of machine learning models using shapley values: Application to compound potency and multi-target activity predictions. J. Comput. Aided Mol. Des..

[47] Ertl P., Rohde B., Selzer P. (2000). Fast Calculation of Molecular Polar Surface Area as a Sum of Fragment-Based Contributions and Its Application to the Prediction of Drug Transport Properties. J. Med. Chem..

[48] Landrum G., Tosco P., Kelley B., Rodriguez R., Cosgrove D., Vianello R., Sriniker, Gedeck P., Jones G., Kawashima E. (2025). rdkit/rdkit.

[49] Friedman J.H. (2001). Greedy function approximation: A gradient boosting machine. Ann. Stat..

[50] Sheridan R.P. (2015). The Relative Importance of Domain Applicability Metrics for Estimating Prediction Errors in QSAR Varies with Training Set Diversity. J. Chem. Inf. Model..

[51] Scalia G., Grambow C.A., Pernici B., Li Y.-P., Green W.H. (2020). Evaluating Scalable Uncertainty Estimation Methods for Deep Learning-Based Molecular Property Prediction. J. Chem. Inf. Model..

[52] Qin M., Sun Z., Feng L., Han C., Xia J., Han L. (2025). MoleculeFormer is a GCN-transformer architecture for molecular property prediction. Commun. Biol..

[53] Cai H., Zhang H., Zhao D., Wu J., Wang L. (2022). FP-GNN: A versatile deep learning architecture for enhanced molecular property prediction. Brief. Bioinform..

[54] Daina A., Michielin O., Zoete V. (2017). SwissADME: A free web tool to evaluate pharmacokinetics, drug-likeness and medicinal chemistry friendliness of small molecules. Sci. Rep..

[55] Daina A., Zoete V. (2016). A BOILED-Egg to Predict Gastrointestinal Absorption and Brain Penetration of Small Molecules. ChemMedChem.

[56] McNemar Q. (1947). Note on the Sampling Error of the Difference Between Correlated Proportions or Percentages. Psychometrika.

[57] Weininger D. (1988). SMILES, a chemical language and information system. 1. Introduction to methodology and encoding rules. J. Chem. Inf. Comput. Sci..

[58] Gaulton A., Bellis L.J., Bento A.P., Chambers J., Davies M., Hersey A., Light Y., McGlinchey S., Michalovich D., Al-Lazikani B. (2012). ChEMBL: A large-scale bioactivity database for drug discovery. Nucleic Acids Res..

[59] Mak L., Marcus D., Howlett A., Yarova G., Duchateau G., Klaffke W., Bender A., Glen R.C. (2015). Metrabase: A cheminformatics and bioinformatics database for small molecule transporter data analysis and (Q)SAR modeling. J. Cheminform..

[60] Kansy M., Senner F., Gubernator K. (1998). Physicochemical High Throughput Screening: Parallel Artificial Membrane Permeation Assay in the Description of Passive Absorption Processes. J. Med. Chem..

[61] Fourches D., Muratov E., Tropsha A. (2010). Trust, But Verify: On the Importance of Chemical Structure Curation in Cheminformatics and QSAR Modeling Research. J. Chem. Inf. Model..

[62] Wildman S.A., Crippen G.M. (1999). Prediction of Physicochemical Parameters by Atomic Contributions. J. Chem. Inf. Comput. Sci..

[63] Lovering F., Bikker J., Humblet C. (2009). Escape from Flatland: Increasing Saturation as an Approach to Improving Clinical Success. J. Med. Chem..

[64] Dietterich T.G. (2000). Ensemble Methods in Machine Learning. Multiple Classifier Systems.

[65] Pedregosa F., Varoquaux G., Gramfort A., Michel V., Thirion B., Grisel O., Blondel M., Müller A., Nothman J., Louppe G. (2018). Scikit-learn: Machine Learning in Python. arXiv.

[66] Varma S., Simon R. (2006). Bias in error estimation when using cross-validation for model selection. BMC Bioinform..

[67] Harris C.R., Millman K.J., van der Walt S.J., Gommers R., Virtanen P., Cournapeau D., Wieser E., Taylor J., Berg S., Smith N.J. (2020). Array programming with NumPy. Nature.

[68] McKinney W. (2010). Data Structures for Statistical Computing in Python. Proceedings of the 9th Python in Science Conference, Austin, TX, USA, 28 June–3 July 2010.

[69] Virtanen P., Gommers R., Oliphant T.E., Haberland M., Reddy T., Cournapeau D., Burovski E., Peterson P., Weckesser W., Bright J. (2020). SciPy 1.0: Fundamental algorithms for scientific computing in Python. Nat. Methods.

[70] Hunter J.D. (2007). Matplotlib: A 2D Graphics Environment. Comput. Sci. Eng..

[71] Waskom M.L. (2021). seaborn: Statistical data visualization. J. Open Source Softw..

[72] Hammarlund-Udenaes M., Fridén M., Syvänen S., Gupta A. (2008). On The Rate and Extent of Drug Delivery to the Brain. Pharm. Res..

[73] An H., Fang J., Wang M., Lin H., Sun Y., Hu B., He Z., Ge Z., Wei Y. (2023). Stereoselective study of fluoxetine and norfluoxetine across the blood–brain barrier mediated by organic cation transporter 1/3 in rats using an enantioselective UPLC-MS/MS method. Chirality.

[74] Montanari F., Zdrazil B., Digles D., Ecker G.F. (2016). Selectivity profiling of BCRP versus P-gp inhibition: From automated collection of polypharmacology data to multi-label learning. J. Cheminform..

[75] Gynther M., Laine K., Ropponen J., Leppänen J., Mannila A., Nevalainen T., Savolainen J., Järvinen T., Rautio J. (2008). Large Neutral Amino Acid Transporter Enables Brain Drug Delivery via Prodrugs. J. Med. Chem..

[76] Angelopoulos A.N., Bates S. (2021). A Gentle Introduction to Conformal Prediction and Distribution-Free Uncertainty Quantification. arXiv.

[77] Sheridan R.P. (2013). Time-Split Cross-Validation as a Method for Estimating the Goodness of Prospective Prediction. J. Chem. Inf. Model..

